# Stiffening Organic Crystals through Polymerization Using Visible Light

**DOI:** 10.1021/jacs.5c15122

**Published:** 2025-10-25

**Authors:** Linfeng Lan, Yuxing Zhou, Liang Li, Chenguang Wang, Panče Naumov, Hongyu Zhang

**Affiliations:** † State Key Laboratory of Supramolecular Structure and Materials, College of Chemistry, 12510 Jilin University, Changchun 130012, P. R. China; ‡ State Key Laboratory of Integrated Optoelectronics, College of Electronic Science and Engineering, 12510 Jilin University, Changchun 130012, P. R. China; § Smart Materials Lab, 167632 New York University Abu Dhabi, PO Box 129188, Abu Dhabi, United Arab Emirates; ∥ SAFIR Novel Materials Development Lab, Sorbonne University Abu Dhabi, PO Box 38044, Abu Dhabi, United Arab Emirates; ⊥ Center for Smart Engineering Materials, 167632 New York University Abu Dhabi, PO Box 129188, Abu Dhabi, United Arab Emirates; # Research Center for Environment and Materials, Macedonian Academy of Sciences and Arts, Bul. Krste Misirkov 2, Skopje MK-1000, Macedonia; ∇ Molecular Design Institute, Department of Chemistry, 5894 New York University, 100 Washington Square East, New York, New York 10003, United States

## Abstract

Soft organic crystals that combine high strength and toughness are essential for flexible electronics and bioinspired devices, but they often compromise one property for the other. Here, we demonstrate a visible-light–driven, single-crystal-to-single-crystal photopolymerization of 1,1′-dioxo-1*H*,1′*H*-[2,2′-biindene]-3,3′-diyl-bis­(decanoate) (**B10**) into a polymeric crystal (**PB10**) that simultaneously with polymerization enhances its mechanical strength and toughness. Under white-light irradiation (2.5 W cm^–2^), centimeter-long **B10** needles exhibit splitting, coiling, and straightening, accompanied by a color change from red to colorless. This transformation is accompanied by a molecular reorganization, where the weak (π···π stacking) interactions are replaced by stronger (C–C) bonds, resulting in a drastic change in mechanical properties. As a result, upon photopolymerization, the **PB10** crystals transition from purely elastic to elastic/plastic, with a nearly 228-fold increase in toughness. This polymerization is also accompanied by increases in tensile modulus and a nearly 81-fold increase in tensile toughness. Remarkably, the **PB10** crystals exhibit a load-bearing capacity exceeding 1 × 10^5^ times their own mass, additionally reflecting the dramatic enhancement in mechanical strength.

## Graphical abstract

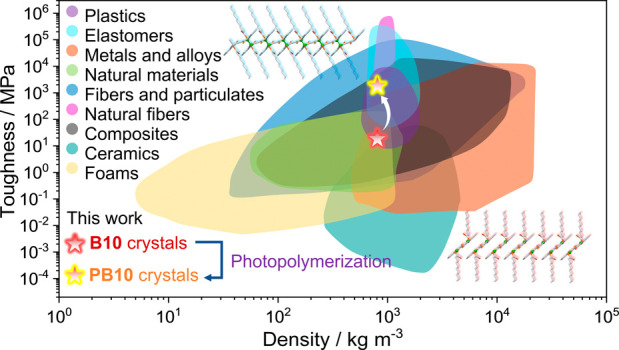

## Introduction

Soft, yet mechanically strong, tough and durable materials are the pillar of advanced technologies such as foldable optoelectronics, implantable/wearable/digestible sensors, self-repairing materials, and adaptive or evolving bioinspired devices.^1–3^ The most immediate advantages of soft organic materials over common inorganic materials, such as those used in the silicon-based (opto)­electronics include their low density and lightweight, recoverable shape and size, favorable biocompatibility, and the potential for both minor and substantial chemical modifications.^4,5^ These traits place them at the forefront of contemporary advanced materials research. While organic materials are in many ways complementary, and in some aspects possibly superior to inorganic materials, one persistent challenge is to develop strategies that effectively harness their mechanical strength and flexibility.^6,7^ Mechanical strength, flexibility, and toughness are interrelated yet distinct mechanical properties. Mechanical strength refers to the maximum stress a material can withstand before failure, flexibility describes the ability to undergo elastic or reversible deformation without breaking, and toughness quantifies the total energy absorbed before fracture, integrating both strength and deformability. The mechanical strength of macromolecular organic materials capitalizes on the high dissociation energy of the strongest interactions in their structure, namely covalent bonds, which can be further enhanced by synergistic electronic effects of multiple strong intermolecular interactions and/or by cross-linking that increases overall strength or reinforces molecular entanglement. However, while they may be readily accessible, organic materials, and specifically the newly researched class of organic molecular crystals, are thought to come with compromised mechanical compliance, and their flexibility and strength appear to be orthogonal and mutually exclusive properties.^8^ The basic mechanical properties of these materials could be additionally altered by exposure to light, heat or mechanical force.^9–13^ Photochemistry, for example, has contributed tools for spatial and temporal control of their crystal structure, and comes with operational convenience, thereby appealing as a promising strategy to “tune” the photophysical properties of organic materials.^14–18^ By harnessing light-induced processes, one can not only manipulate the molecular packing of organic crystals, but also readily trigger macroscopic dynamic responses that in kinetics can range from very slow to extremely fast processes.^19–32^

While topochemical photopolymerization has proven effective in enhancing the mechanical properties of bulk polymers and polycrystalline materials, its translation to single-crystal systems is not straightforward.^33–37^ In particular, achieving simultaneous improvements in strength, flexibility, and toughness without compromising crystallinity presents a formidable challenge.^38–42^ Here, we present a case in which the mechanical toughness of an organic crystal is significantly improved through photopolymerization. Through a two-step process, crystals of 1,1′-dioxo-1*H*,1′*H*-[2,2′-biindene]-3,3′-diyl-bis­(decanoate) (**B10**; Figure 1a) undergo splitting and curling, followed by gradual straightening and color change. This transformation results in the formation of a solid mixture of polymers (**PB10**), which has strength, flexibility, and toughness that are many times higher relative to that of the reactant crystal. Photopolymerization replaces the weak π···π interactions in the monomer with strong covalent C–C bonds, preserving the crystal lattice and enhancing mechanical robustness through topochemical transformation. Simultaneously, the single-crystal-to-single-crystal nature of this polymerization allows the material to maintain its flexibility in crystalline form. This work provides a pathway for the design of flexible, yet mechanically robust high-performance polymeric organic materials via simple solid-state photochemistry. Within a more general context, it demonstrates the capacity of light-induced structural transformations, which have been well-known from the solid-state organic chemistry perspective, to change and possibly to control one of the most essential properties of organic crystalline materials, namely their mechanical robustness.

**Figure 1:**
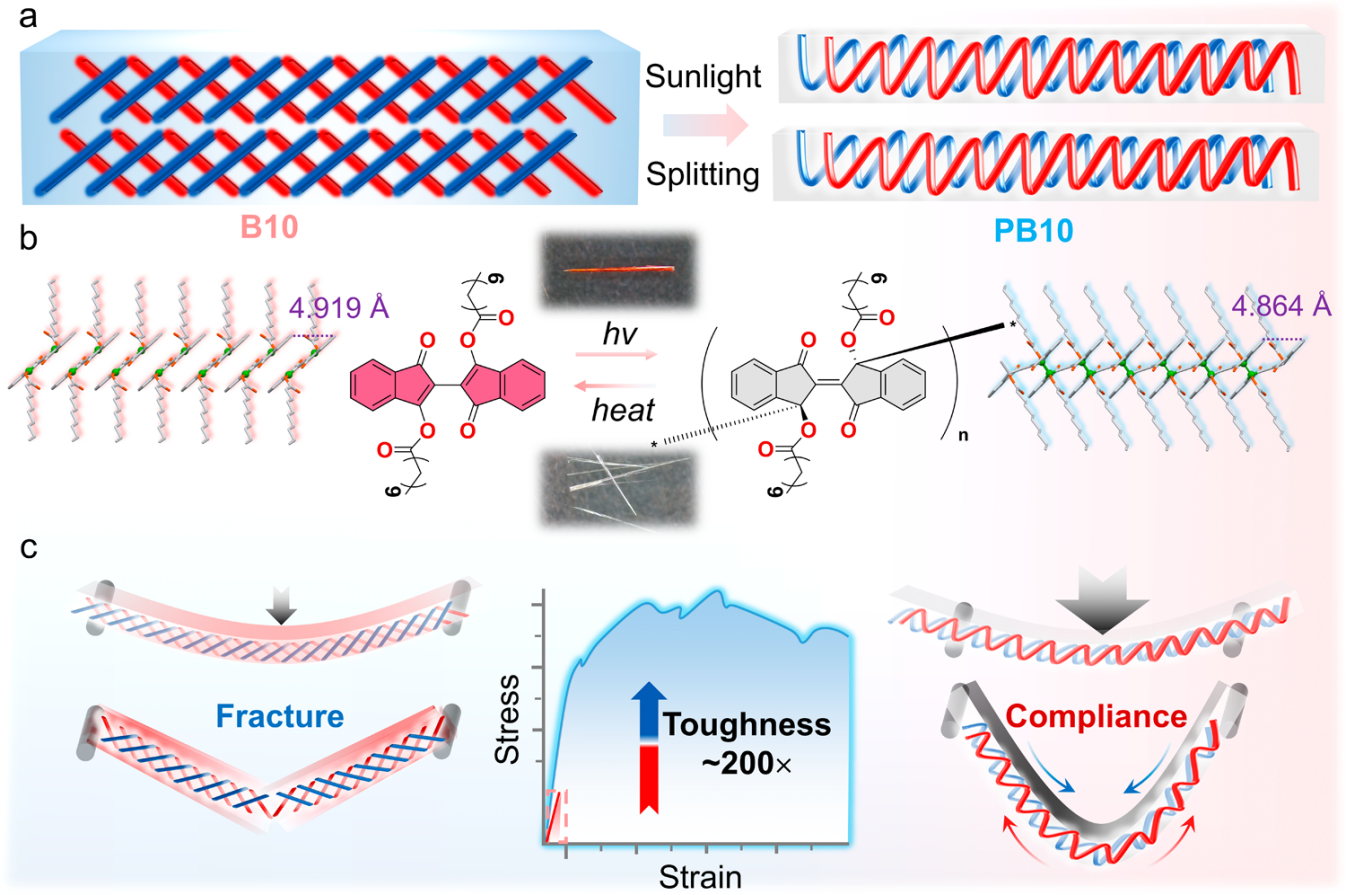
Visible-light-induced single-crystal-to-single-crystal transformation enhances the mechanical toughness of an organic crystalline material. (a) Schematic showing the polymerization of an ordered single crystal into linear polymer chains with natural sunlight. (b) Photochemical transformation from stacked monomer units in **B10** to covalently linked linear polymer chains in **PB10**. Inset: optical images of the original crystal **B10** (top) and the photopolymerized crystal **PB10** (bottom). (c) Schematic representation of the correlation between structure and mechanical strength, illustrating the observed significant enhancement in toughness and the mechanism postulated for this observation.

## Results and Discussion

The molecule of the monomer, **B10**, was synthesized following a procedure described earlier (Scheme S1, Figures S1 and S2).^43,44^ Crystals of the monomer were prepared by dissolving **B10** in dichloromethane, followed by the addition of ethanol, and slow evaporation at 278 K in the dark. As shown in Figure 1, a photoreaction between the adjacent **B10** molecules induced by natural sunlight converts the arrays of its molecules into linear chains of polymers that could be composed of a varying number of monomeric units and different molecular weights, in this work jointly referred to as **PB10**. Single-crystal X-ray diffraction analysis confirmed the photochemical transformation that is accompanied by a significant structural change (Figure 1b), as is discussed in detail below. While the weak van der Waals interactions in the **B10** crystals can be readily disrupted by the application of external force, the energetically more stable covalent C–C bonds are more resilient to rupture; we thus anticipated that the polymerization would affect the stiffness and hardness of the reacting crystal (Figure 1c).

The crystals of **B10** exhibit a broad absorption across the entire visible light region and weak red fluorescence with a maximum around 620 nm. The strong absorption facilitates the photoreaction process by enabling efficient light absorption over a wide range of wavelengths (Figure S3a). As shown in Figure 2a, crystals of **B10** are exceptionally dynamic when exposed to visible light and undergo mechanical effects such as directional splitting along the growth axis, curling and entanglement, and uncoiling and straightening, which are crucial for understanding their mechanical response to external stimuli. Under white light (2.5 W cm^–2^), approximately 1 cm-long needle-like **B10** crystals rapidly coil and then jump (Figures 2b, S4a,b and Movie S1). The crystal size affects the dynamic response; shorter, approximately 0.2 cm-long rod-like **B10** crystals of similar width and thickness undergo only partial splitting, but remain static (Figures 2c and S4c,d). Regardless of the size, all crystals ultimately transform into straight, colorless transparent or white (depending on the degree of their opaqueness) polymeric crystals of **PB10** (Figure S3b,c). The temporal evolution of the diffuse reflectance UV/vis absorption spectra during the reaction (Figure S5) shows a gradual decrease in the characteristic absorption bands of the monomer, consistent with the progressive photopolymerization process. Solid-state transmission measurements (Figure S6), performed on hundreds of **B10** crystals, show that the transmittance in the 400–600 nm region steadily increases with irradiation time until the crystals turn completely colorless, indicating that light can penetrate the polymerized surface layers and drive subsurface monomer conversion. The single-crystal-to-single-crystal transformation was also observed under both 365 and 530 nm irradiation, with slight variations in the reaction speed and intermediate crystal morphologies (Figure S7). These results indicate that the transformation can be induced by different excitation wavelengths, consistent with the absorption characteristics of **B10**.

**Figure 2:**
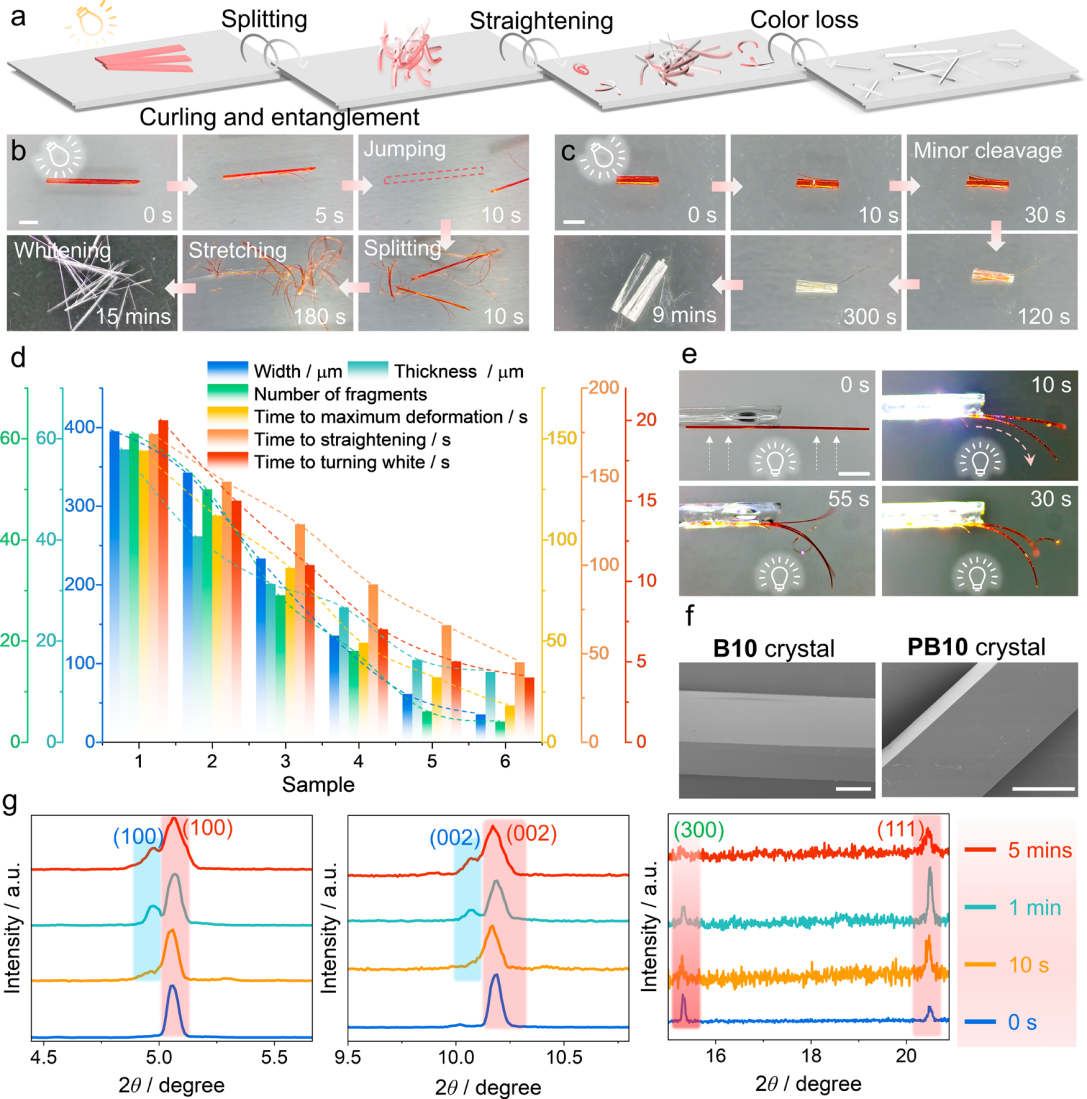
Mechanical effects of dynamic organic crystals of **B10** under external stimuli. (a) Schematic illustration of the mechanical effects (directional splitting, curling, and straightening) and photoinduced color loss observed with the crystals of the monomer when they react under visible light. (b) Sequential optical images showing the jumping of a 1 cm-crystal under white light irradiation (2.5 W cm^–2^) at different time points. (c) Cleavage of a 2 mm-crystal under prolonged exposure to white light. (d) Quantitative analysis of the crystal size (width, thickness) and other parameters used to quantify the mechanical effects. Specifically, these parameters include the number of crystal fibers generated by splitting during the photoreaction, the time required to reach maximum deformation (coiling), the time needed for subsequent straightening of the crystal, and the time it takes for the crystal to turn completely colorless. (e) Bending and curling of a crystal of **B10** in response to exposure to a white light source (2.5 W cm^–2^). (f) Scanning electron microscopy (SEM) images comparing the surface morphology of crystals of **B10** and **PB10**. (g) Relevant regions in the in situ powder X-ray diffraction patterns illustrating the evolution of the polymeric structure during irradiation of PVA-coated **B10**. The scale bar in panels b, e is 2 mm, in panel c it is 1 mm and in panel f it is 50 μm.

Quantitative analysis of crystalline samples with varying widths and thicknesses provided a comprehensive overview of the crystal deformation–recovery kinetics. We found a positive correlation between the crystal size and the extent of cleavage, maximum deformation, straightening, and the time required for the crystal to turn completely colorless (Figure 2d and Table S1). The results suggest that larger crystals undergo more extensive deformation and take a longer time to reach their final shape (Figure S8). We also observed that during the photochemical reaction needle-like crystals break into numerous spiral or spring-like fragments, which are mechanically elastic and can be reversibly bent (Figure S9). Sufficiently thin crystals maintain their single-crystal form without cracking, even when they are bent into a helical shape during the photoreaction (Figure S10). This further supports the hypothesis and demonstrates the remarkable flexibility and mechanical stability of the crystals of the product obtained by light-induced deformation. The bending and splitting of the crystals are driven by light-directed deformation toward the illumination source (Figures 2e, S11 and Movie S1), resulting from a differential strain. The strain is generated by unit cell mismatch due to the decrease in volume when the molecules of the monomer are transformed into those of the polymers (Table S2).

From the unit cell parameters at room temperature, we infer that upon polymerization a crystal of **B10** undergoes a single-crystal-to-single-crystal transformation accompanied by a ∼4% reduction in unit cell volume (from 1695.32(9) to 1627.82(9) Å^3^). The red crystals of **B10** of varying sizes all underwent deformation and turned colorless upon irradiation. We noticed that smaller crystals reacted faster, and the fractured crystals remained transparent and had a smooth surface (Figures 2f, S12 and S13). Comparison of the measured and simulated powder X-ray diffraction (PXRD) patterns (Figure S14) before and after photopolymerization showed shifting of the (100), (200), and (002) peaks to lower 2*θ* values, along with disappearance of the (300) peak and emergence of a new peak, (204), indicating selective reorganization within the crystal structure. Additionally, in situ PXRD analysis was performed on crystals immobilized in poly­(vinyl alcohol) (PVA) to prevent movement of the crystals during the reaction (Figure S15). The photopolymerization in this state was noticeably slower. However, the gradual emergence of new diffraction peaks corresponding to the polymer, including the (100) and (002) peaks, was still observed, accompanied by a progressive disappearance of the original (300) peak (Figure 2g). This indicates that the photopolymerization can also occur and proceed in a spatially confined environment, and that the transformation of the monomers to the polymers still results in a structurally ordered product.

To understand the mechanism of photopolymerization, we analyzed the crystal structures of **B10** and its polymerized form, **PB10**. Both crystals are in the monoclinic space group *P*2_1_/*c*. The photopolymerization induces comparable shrinkage in the unit cell axes *a*, *b*, *c* by 3.41, 1.53, and 1.88%, with a reduction in the β angle from 117.461(6) to 114.088(2)° (Table S2). These changes suggest that photopolymerization results in contraction of the crystal lattice due to the formation of covalent bonds that replace other, much weaker intermolecular interactions. For both **B10** and **PB10** the longest crystal axis is the [010] direction, with the wide face identified as the (100) plane based on face indexing (Figures 3a and S16). In **B10**, the distance between the reactive bonds is *d*_C···C_ = 3.233 Å, and the aromatic rings are coplanar (interplanar angle = 0°). This disposition of the reactive bonds is consistent with the topochemical requirements (distance of 3.5–4.2 Å and near-planarity) for polymerization (Figure 3b).^45,46^ Upon exposure to visible light, the photopolymerization induces a significant molecular reorganization. The angle between the five-membered ring and the benzene ring increases to 27.7°, resulting in a twisted molecular backbone. This twisting facilitates the formation of new C–C bonds, and enhances the rigidity and stability of the resulting polymer network. In the crystal structure of **B10**, weak intramolecular interactions or contacts are observed between the ketone group and the ester group, specifically C–O···C (2.672 Å, 105.3°) and C–O···O (2.658 Å, 121.3°) (Figure 3c). Along the *b*-axis, the adjacent molecules are stabilized by π···π interactions (3.284 Å) and C–H···O hydrogen bonds (C···O: 3.394 Å; H···O: 2.510 Å; ∠C–H···O: 154.9°) (Figure 3d). Along the *a*- and *c*-axes, each molecule interacts with six neighboring molecules via multiple H···H contacts: (1) between the aromatic hydrogen atoms and the central hydrogens of the ester chain (H···H: 2.225 Å), and (2) between the terminal ester-chain hydrogens and aromatic hydrogen atoms from adjacent molecules (H···H: 2.388 Å), which are expected to be more susceptible to disruption during the polymerization process (Figure 3e). In the structure of the product, the intramolecular distances are shortened and no detectable intermolecular interactions exist between the polymer chains, resulting in a more compact molecular packing. The original intermolecular hydrogen bonds are transformed into stronger, intramolecular ones (2.325 Å). In addition to retaining the original intramolecular contacts (C–O···C: 2.685 Å; C–O···O: 2.649 Å), the polymer chains exhibit more extensive interactions between the adjacent aromatic segments (O···O, C···O and C···C distances ranging from 2.564 to 3.363 Å) (Figure 3f). Frontier orbital analysis of **B10** (Figures 3g, S17 and S18) indicates a shift in electron density from the delocalized state in the highest occupied molecular orbital (HOMO) to a more localized state around the C=C double bonds in the lowest unoccupied molecular orbital (LUMO). This electronic shift enhances the reactivity of the polymerization sites, facilitating the breaking of the C=C bonds and promoting chain growth, which is essential for the efficiency and selectivity of the photopolymerization process. Additional calculations on a model dimer indicate that the orientation and symmetry of the frontier orbitals facilitate efficient overlap between the reactive C=C sites, further supporting the promotion of chain growth (Figure S19). Accompanying the structural evolution, the HOMO–LUMO energy gap decreases from 2.95 eV in **B10** to 2.12 eV in **PB10**, indicating a reduced excitation energy barrier.

**Figure 3:**
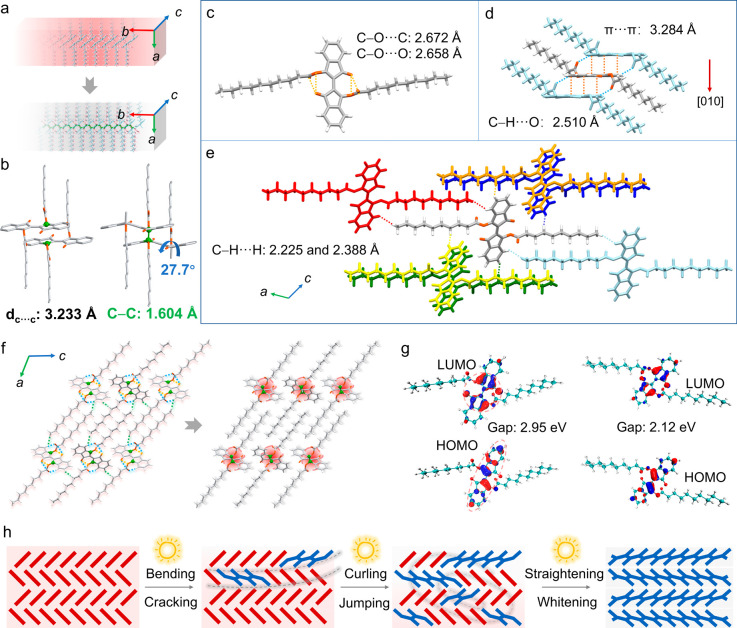
Mechanism of the photopolymerization of **B10** crystals. (a) A schematic representation of the molecular orientation in the unit cells of **B10** and **PB10**. (b) Parallel stacking of **B10** molecules showing the distances between the reactive carbon atoms (left), and the twisted parallel stacking of adjacent **PB10** molecules showing a C–C bond length of 1.604 Å (right). (c–e) Crystal structures of **B10** showing the relevant intramolecular interactions (c) and intermolecular contacts or distances (d,e). (f) Molecular packing shown in the direction of the crystallographic *b*-axis (growth direction) and changes that occur upon photopolymerization. The blue, green, and yellow dashed lines indicate the C–H···O, C–H···H, and C–O···C/O distances, respectively. (g) Frontier orbital analysis of the original **B10** (left) and **PB10** (right) crystals. (h) Proposed mechanism for the photomechanical effects based on the crystal structures.

Based on these observations, the mechanism of photopolymerization can be summarized as follows (Figure 3h): upon light exposure, the reaction occurs in regions near the light source and affords a mixture of polymeric products of various lengths, depending on the extent of the chemical reaction. The smaller volume of the product compared to the ensemble of the reacting monomers results in shrinking of the crystal close to the surface, in the regions where the product is generated. The process results in the development of internal differential strain that induces bending of the crystal. Occasionally, it is accompanied by evolution of cracks and splitting of the crystal. These effects collectively lead to the observed pronounced deformation of crystal filaments and result in reshaping of the crystals into coils, helices, and other shapes. As the polymerization advances, the deformation reaches a mechanical equilibrium; once the fraction of the polymer chains surpasses a threshold, the elasticity of the polymer becomes dominant, and induces gradual straightening of the crystal.

The photoreaction is accompanied by a change of the color from orange-red to colorless, and the reaction progress can be monitored from the UV–vis absorption spectra (Figure S20). To quantify the extent of polymerization, 100 mg crystals of **B10** were irradiated with white light for 2 h, and thoroughly washed with dichloromethane to remove any unreacted monomer. After drying, the remaining mass was 95 mg (Figure S20), indicating a polymerization degree of approximately 95%. This result is consistent with the thermogravimetric analyses of the monomer and polymerized crystals, which both show single-step decomposition behavior, confirming the uniform structure of the polymerized crystals and supporting the high polymerization degree (Figure S21). As the crystals are irradiated, they start to split, entangle with each other, and form a three-dimensional network (Figure S22). The molecular rearrangement required for complete polymerization, and thus for the full color change from orange-red to white, must occur throughout the bulk of the crystal. As a result, the color change proceeds more slowly than the mechanically induced deformation observed at the surface. Due to the diffusion-limited reaction and gradual propagation of the polymerization front through the crystal bulk, similar delayed optical responses during photopolymerization have been observed in other topochemical systems, where the mechanical strain precedes the changes monitored by spectroscopic or other methods.^47,48^

Upon heating to approximately 483 K, the colorless solid of **PB10** gradually converts back to the red **B10**, which subsequently begins to melt (Figure S23a and Movie S2). This is evidenced by the differential scanning calorimetry results, where a broad depolymerization transition appears first, followed by a shoulder peak at 488 K, which likely corresponds to the melting of the polymer fraction or partially reorganized structures formed during the depolymerization process (Figure S24). This sequence confirms that thermal depolymerization of **PB10** to **B10** occurs prior to melting, rather than the melting triggering the depolymerization. The crystal of the pure monomer **B10** has a distinct melting point at 368 K, further supporting this interpretation. No crystallization peaks were observed above this transition, indicating the formation of an amorphous state. This conclusion is further supported by the PXRD and UV–vis spectra of the solid recorded before and after heating (Figure S25a–c). The changes in the diffraction pattern and the reappearance of absorption features characteristic of the **PB10** crystals, partially depolymerized crystals (heated to 483 K), and the fully melted and resolidified solids (heated to 488 K) confirm the occurrence of depolymerization, regeneration of monomers, and loss of long-range order. Additionally, the ^1^H NMR spectrum of the thermally treated sample matches that of the pristine monomer, confirming the chemical integrity of the depolymerized species (Figure 25d). The absence of peaks in the second heating cycle is consistent with the loss of both crystallinity and photoreactivity, as the resulting amorphous monomer can no longer undergo photopolymerization upon light exposure (Figures S23–S25). Heating the **B10** crystals alone to 353 K does not induce any photopolymerization, while simultaneous heating and light exposure promote the reaction to some extent (Figures S23b, S26). Interestingly, the crystals of **B10** do not undergo photopolymerization under white light at low temperatures, likely due to restricted molecular motion that suppresses the photochemical reaction (Figure S27). Together, these findings confirm that temperature has a significant impact on this photopolymerization reaction, as it has been concluded previously.^49^

The structural changes induced by the photopolymerization, based on the crystal structures, correlate with the enhancement of mechanical properties observed upon conversion of **B10** to **PB10** crystals (Figure 4a). At room temperature, the crystals of **B10** exhibit good elasticity and are capable of reversible deformation after they have been bent (Figures 4b, S28 and Movie S3). The maximum elastic strain (ε) before fracture was estimated to be 1.81 ± 0.22% based on the maximum curvature of the bent crystals and the Euler–Bernoulli beam-bending theory.^50^ In contrast, **PB10** crystals demonstrate superior elastic bending performance, achieving ε = 3.85 ± 0.41% before undergoing significant irreversible plastic deformation (Figures 4c, S29 and Movie S3). Further comparison of their mechanical properties at low temperatures reveals that crystals of **B10**, when placed on a silicon substrate at 123 K or immersed in liquid nitrogen maintain remarkable flexibility, bending significantly in both directions (Figures 4d, S30 and Movie S4). Similarly, crystals of **PB10** retain both elasticity and plasticity at low temperatures, with the elastic bending region remaining intact even in sections undergoing plastic deformation, demonstrating compatibility between these mechanical behaviors (Figures 4e, S31 and Movie S4). Based on the analysis of the bending limits of both crystals at room and low temperatures, **PB10** can withstand about twice the fracture strain of **B10** under both conditions; at 298 K, ε = 1.86 ± 0.22% vs 3.85 ± 0.41%, and at 123 K ε = 1.61 ± 0.10% vs 3.49 ± 0.41%. These results highlight the increased elasticity and deformation capacity of the polymerized crystal (Figure 4f,g). The enhanced mechanical robustness is due to the polymeric structure, which improves the ability of the crystal to absorb and dissipate stress.

**Figure 4:**
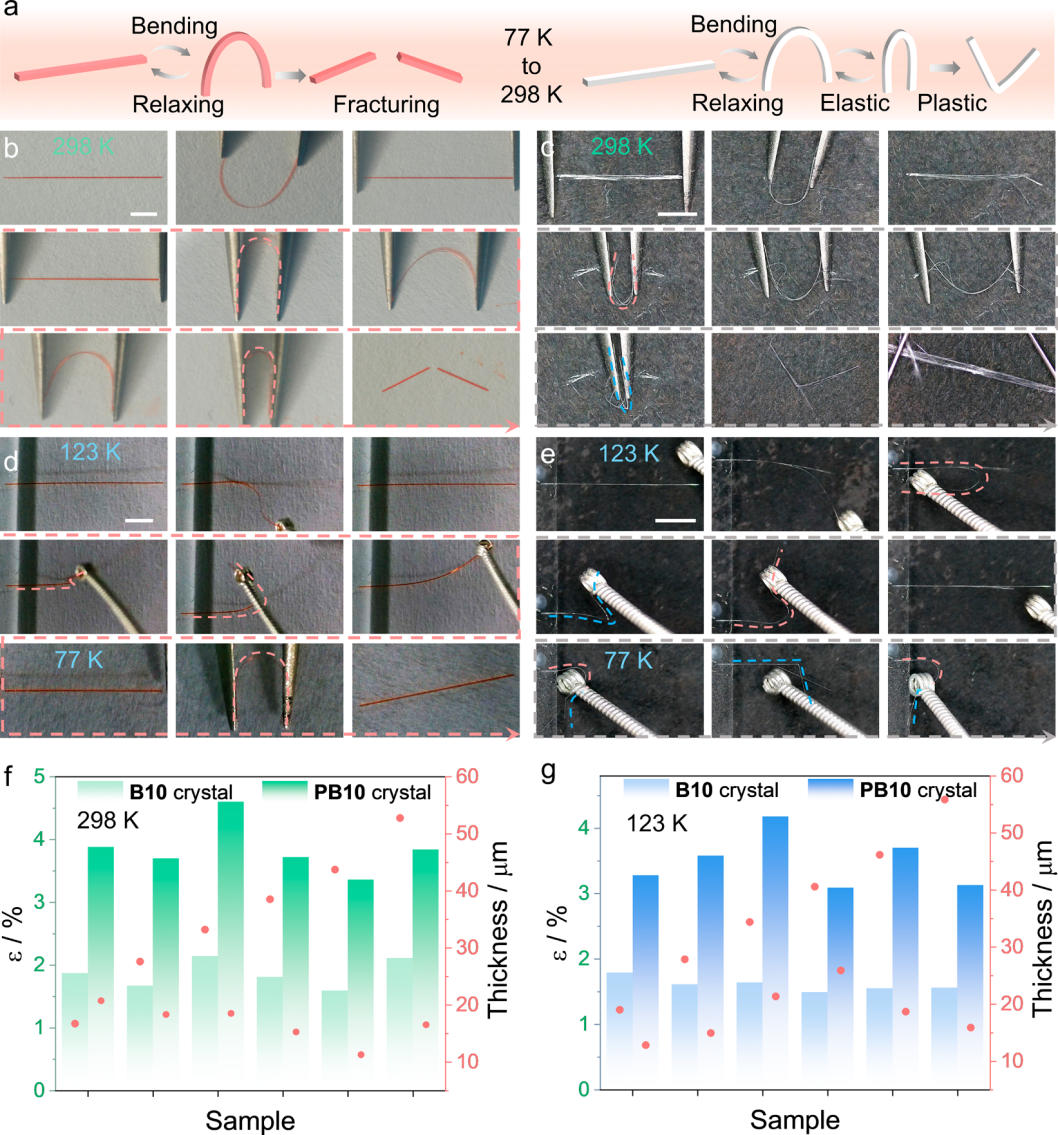
Comparison of the mechanical properties of crystals of the monomer **B10** and the polymer **PB10**. (a) Schematic comparison of the elastic and plastic behavior of **B10** and **PB10** crystals. (b,d) Reversible bending of **B10** at room temperature (b) and at low temperature, such as liquid nitrogen (d). (c,e) Elastic bending and plastic deformation of **PB10** at room temperature (c) and at low temperature (e). (f,g) Comparison of the maximum elastic strain (ε) and thickness of **B10** and **PB10** crystals at room temperature (f) and at low temperature (g). The length of the scale bar in panels b and c is 2 mm.

The differences in the macroscopic mechanical properties of **B10** and **PB10** can be attributed to changes in molecular packing and arrangement. The key structural transformation is the replacement of the loosely associated molecules of the monomer that interact by π···π stacking to covalently bonded units in the polymer chains (Figure 5a), which leads to notable enhancement in the mechanical properties of **PB10**. Figure 5b,c depict the molecular arrangements in the original and polymerized crystals viewed along different axes. In the direction of both the *c*-axis and *a*-axis, the molecules in **B10** maintain a parallel stacking pattern. We hypothesize that when external force is applied, the energy is dissipated by breaking and reorganization of the intralayer π···π interactions and the interlayer van der Waals forces, and this structural perturbation aids the crystal in maintaining a certain level of mechanical flexibility. Energy framework calculations show significant anisotropy in the interactions in the structure of **B10**. Along the crystallographic *b* axis, strong π···π and C–H···O interactions dominate (111.8 kJ mol^–1^), while along the *a* and *c* axes, the interactions (−14.8 to −45.5 kJ mol^–1^) are much weaker and mostly C–H···H contacts (Figure S32). After photopolymerization to **PB10**, the stacking pattern remains unchanged, and supports the elasticity of the product. Additionally, the flexible alkyl chains at both ends of the molecule not only further facilitate deformation, contributing to the overall structural mechanical adaptability and stability of the crystal, but they also form potential slip planes along the *a* and *c* axes. With only weak interactions between the chains in **PB10**, the highly aligned polymer chains are expected to misalign upon further bending, resulting in macroscopic plastic deformation, evolution of defects, and possibly even splitting.

**Figure 5:**
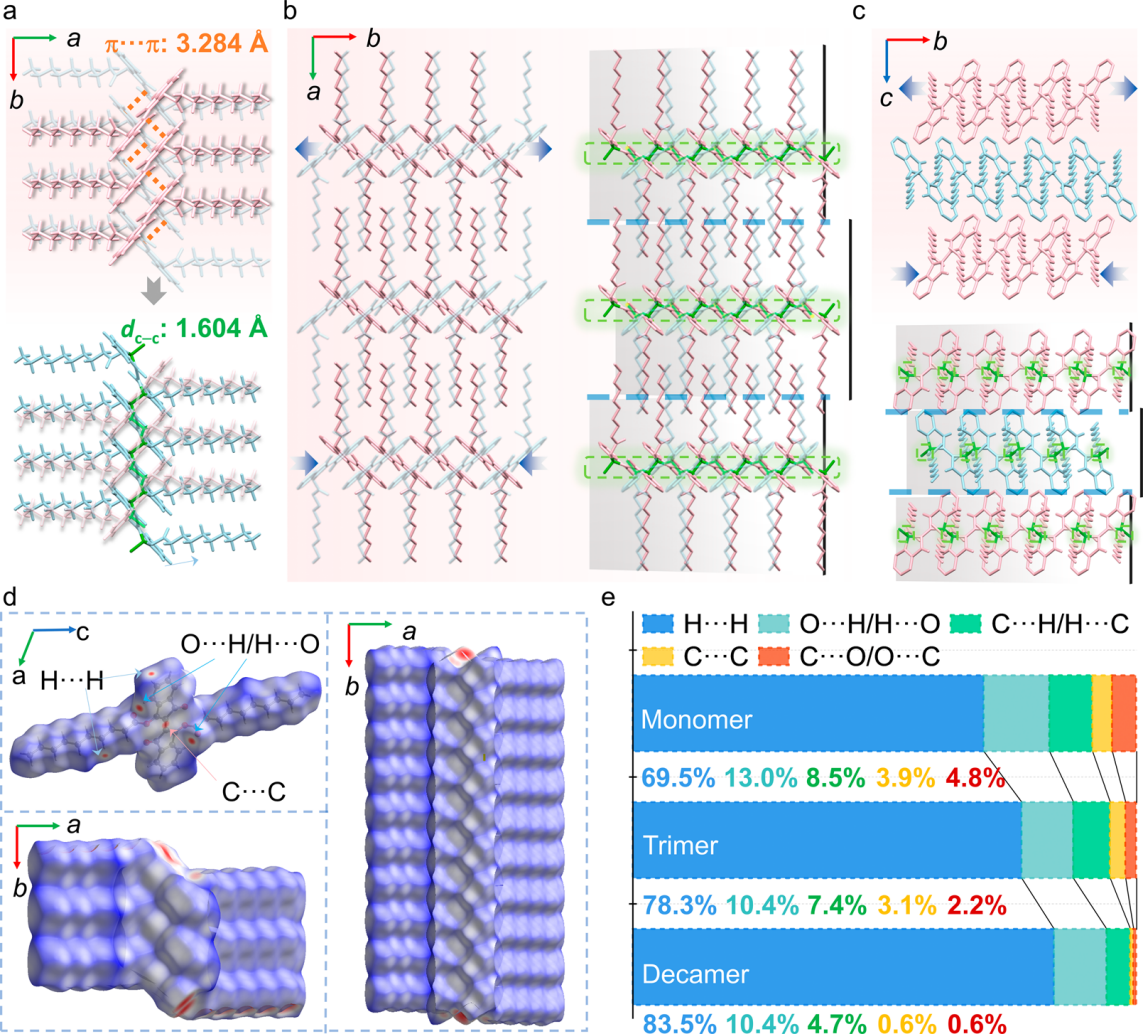
Evolution of the structure of a single crystal of **B10** upon polymerization. (a) Replacement of the dominant interaction, π···π stacking (orange dotted line) in **B10** with C–C single bonds (green line) in **PB10** along the [010] direction. (b) Molecular packing in the original (left) and polymerized (right) crystals, viewed in the direction of the *c* axes in the respective structures. (c) Molecular packing in the original (top) and polymerized (bottom) crystals, viewed in the direction of the *a* axes in the respective structures. The green areas highlight the polymer chain segments, and the blue dashed lines indicate the possible slip plane. (d) Hirshfeld surface analysis of the monomer in the structure of the **B10** crystal, and trimer and decamer fragments from the **PB10** crystal. (e) Bonding contributions, based on the Hirshfeld analysis, of the most relevant intermolecular contacts in the crystals.

Upon cooling, the unit cells of both **B10** and **PB10** undergo slight, relatively isotropic shrinkage that strengthens the intermolecular interactions, yet their stacking pattern remains unchanged, which explains their excellent flexibility at low temperatures (Figure S33 and Table S3). Hirshfeld surface analysis performed for the monomer in the **B10** crystal and for trimer and decamer fragments extracted from the structure of the **PB10** crystal (Figures 5d and S34–S36) shows that the proportion of H···H interactions increases from 69.5 to 78.3% and then to 83.5%, while the proportions of hydrogen bonds, C···H/H···C and π···π interactions decrease (Figure 5e). This change in bond contributions enhances the molecular packing density, reduces structural defects, and leads to more isotropic distribution of intermolecular interactions, highlighting the critical role of interaction reorganization in photopolymerized material, as has been demonstrated with other solid-state polymerizations.^51,52^

Building on the structural insights discussed above, the mechanical toughness of the polymerized crystal **PB10** was compared to that of a crystal of the reactant **B10**. When punctured with a needle tip, crystals of **B10** tend to fracture (Figure 6a,b and Movie S5). In contrast, the crystals of the polymer **PB10** do not shatter, but instead split along their long axis into multiple slender fibrils, demonstrating significantly enhanced ductility relative to the monomer (Figure 6b). Atomic force microscopy images reveal decreased surface roughness upon polymerization, from *R*_a_ = 13.6 nm for **B10** to *R*_a_ = 2.60 nm for **PB10**, and show that the polymerization results in a smoother surface (Figure S37a,b). Based on the load–displacement curves obtained by nanoindentation, **PB10** has elastic modulus (based on nanoindentation) *E* = 3.014 ± 0.354 GPa and hardness *H* = 0.305 ± 0.022 GPa, compared to *E* = 1.072 ± 0.190 GPa and *H* = 0.135 ± 0.019 GPa for **B10**, in line with the substantial stiffening and strengthening of the crystal upon polymerization (Figure S37c,d).

**Figure 6:**
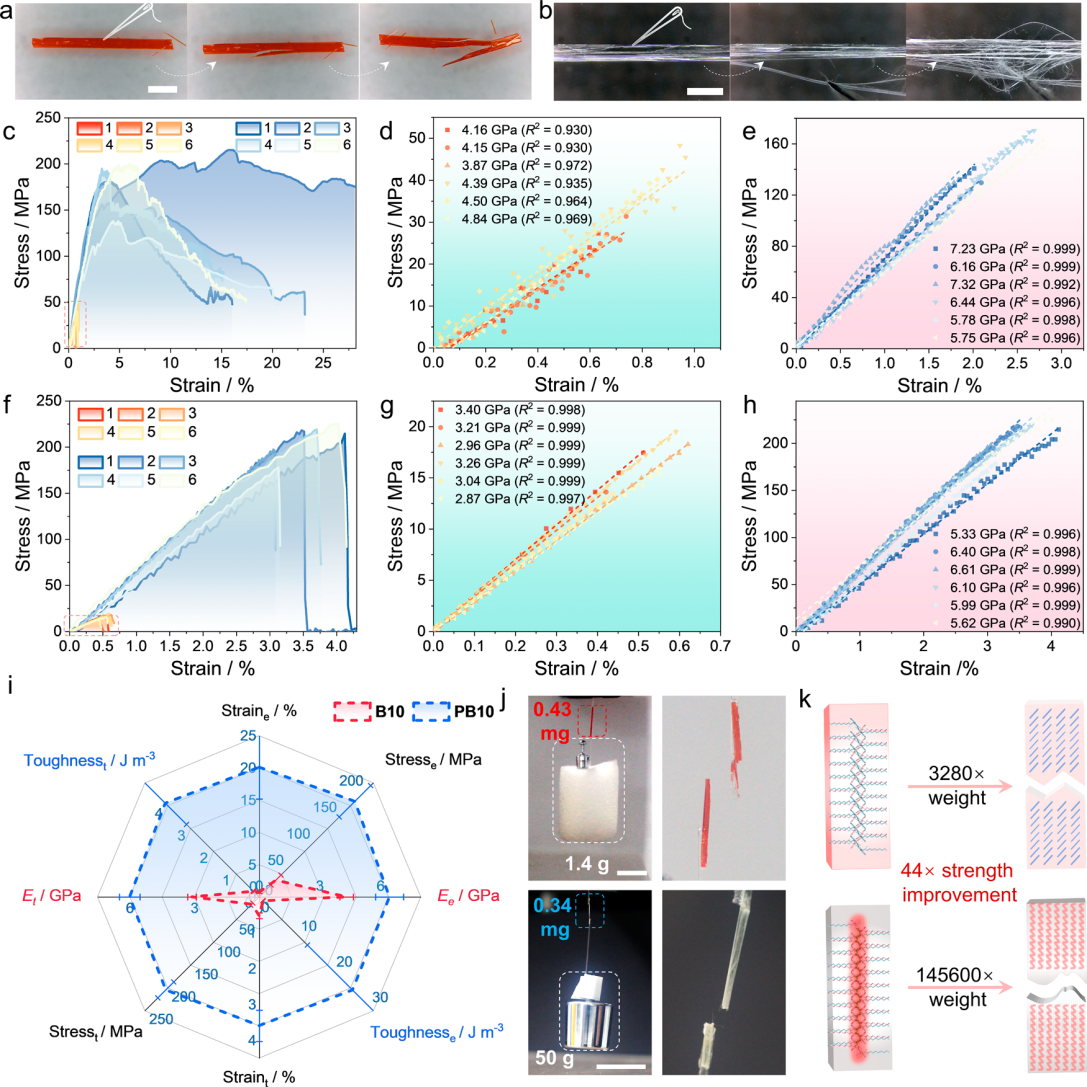
Enhancement of mechanical robustness by polymerization. (a,b) Photographs of **B10** (a) and **PB10** (b) crystals before and after they have been pinched by a sharp tip (diameter 120 μm). (c–e) Stress–strain curves (c) and fitting to estimate the elastic modulus (*E*_e_) of **B10** (d) and **PB10** (e) crystals determined by three-point bending tests. (f–h) Stress–strain curves (f) and fitting to estimate the tensile modulus (*E*_t_) during tension of **B10** (g) and **PB10** (h) crystals, demonstrating the polymerization-induced increase in ductility and tensile strength. The modulus, extracted and compared across different samples, highlights the superior flexibility and toughness of **PB10** relative to **B10**. (i) Radar chart comparing the overall mechanical performance of **B10** and **PB10**, revealing a significant improvement in performance of **PB10** relative to that of crystals of the monomer **B10** across all metrics. (j) Demonstration of the ability of a single crystal and a polymer crystal to perform work, by utilizing crystals of **B10** and **PB10** to lift a weight. (k) Schematic illustration of the strength comparison, showing the enhanced structural integrity and strength of **PB10**. The length of the scale bar in panels a, b is 2 mm, and in panel j is 1 cm.

The mechanical properties of **B10** and **PB10** were further evaluated using three-point bending tests (Figure S38 and Movie S6). Due to the tendency of the crystals for splitting during photopolymerization, large crystals of **B10** were first coated with PVA and then photopolymerized. The PVA coating was peeled off under hot water (Figure S39), and the **PB10** crystals were analyzed by three-point bending. The stress–strain curves confirmed the linearly elastic behavior of **B10** and the plasticity of **PB10** (Figure S38). The polymerization increases both the ultimate strain and fracture strength, demonstrating enhanced flexibility of the crystal (Figure 6c–e). The elastic modulus (based on three-point bending) increased from *E*_e_ = 4.32 ± 0.33 GPa to 6.45 ± 0.69 GPa, while the toughness increased significantly, from *U*_e_ = 0.11 ± 0.06 J m^–3^ to 25.14 ± 12.60 J m^–3^ (Table S4). This change represents an impressive 228-fold increase in toughness, which plays a crucial role in enabling the material to absorb and distribute external stresses effectively. Additional tests conducted on different sites of a **PB10** single crystal showed similar toughness and elastic modulus, confirming the uniformity of these properties throughout the crystal (Figure S40). Tensile testing further confirmed the photopolymerization-induced enhancement in mechanical properties (Figure S41 and Movie S7). Stress–strain curves and tensile modulus fitting revealed that **PB10** has favorable ductility and tensile strength (Figure 6f–h). Specifically, the tensile modulus *E*_t_, which is close to the *E*_e_, increased from *E*_t_ = 3.12 ± 0.20 to 5.84 ± 0.74 GPa, and the toughness *U*_t_ increased significantly from *U*_t_ = 0.049 ± 0.013 to 3.99 ± 0.95 J m^–3^, which represents an approximately 81-fold improvement (Table S5). The global materials property plots of toughness versus density and bending fracture strength versus tensile fracture strength clearly demonstrate the significant enhancement in mechanical performance (comparative data were obtained using the Granta Selector 2024, ANSYS; Figures S42 and S43).

The radar chart in Figure 6i provides an integrated comparison of overall mechanical performance, highlighting the superior mechanical properties of **PB10** across all tested parameters. To better illustrate the differences in strength, **B10** and **PB10** crystals of similar size were selected for simple experiments aimed at demonstrating the work-performing capacity of these materials (Figure S44a and Table S6). A 0.427 mg-crystal of **B10** could lift only 1.4 g of total weight, but it fractured when the weight increased to 2.4 g (Figures 6j, S44b,c and Movie S8). In contrast, a 0.343 mg-crystal of **PB10** could easily lift 12.5, 20, 40, and 50 g in succession, but it fractured at 100 g (Figures 6j, S44d, S45 and Movie S8). Even at a qualitative level, these experiments demonstrate that the polymerized crystals can bear objects tens of thousands of times their weight, showcasing a significant improvement in the material strength. Additionally, while the original crystal was prone to shear or torsional failure (Table S7), **PB10** crystals withstood torsional deformation under a 17.5 g load without fracturing, in further support of their superior torsional stability (Figure S46). Additional tests on individual crystals confirmed the reproducibility of this enhancement. **B10** crystals with masses of 0.046–0.293 mg lifted 0.4–1 g (weight ratios ∼2800–3400), while **PB10** crystals with masses of 0.047–0.135 mg lifted 8–25 g (weight ratios >1.3 × 10^5^), highlighting the dramatic increase in load-bearing capacity after polymerization (Table S8). Overall, the results indicate that polymerization significantly improves the mechanical toughness of the crystalline material, primarily by increasing ductility, reducing brittleness, and enhancing stress distribution capability (Figure 6k). Furthermore, when subjected to pressure while clamped between glass slides, **B10** crystals exhibit progressive failure under low loads (1–5 N) that ultimately results in complete fracture (Figure S47a). In contrast, the **PB10** crystals maintain structural integrity under applied loads up to approximately 100 N, displaying only moderate plasticity, and eventually split when the load reaches about 200 N (Figure S47b). This improvement further underscores the pivotal role of polymerization in reinforcing the crystal structure and enhancing its mechanical robustness.

## Conclusions

In this study, we have demonstrated that a visible-light-driven photopolymerization significantly enhances the mechanical toughness of an organic crystal. Through a single-crystal-to-single-crystal transformation, the crystal of the monomer undergoes a light-induced transformation involving mechanical deformation and a distinct color change, which leads to the formation of a polymeric structure with significantly enhanced mechanical properties, including greater strength, ductility, and toughness. The photopolymerization mechanism, which involves a transition from π···π stacking to C–C single bonds, is responsible for the enhanced mechanical robustness. Our results show that **PB10** outperforms **B10** in several mechanical tests, including bending, tensile, and fracture tests, highlighting the potential of this strategy for designing flexible and durable organic materials. Additionally, the ability to control the photopolymerization process with visible light provides an environmentally friendly and versatile approach to modulating the properties of organic crystals. These findings open up new possibilities for the development of high-performance organic materials for a wide range of applications, from flexible electronics to bioinspired devices. This work also provides valuable insights into the relationship between the molecular structure and mechanical performance in organic crystals, offering guidance for future efforts to design materials with tailored mechanical properties. Moving forward, we foresee that the development of new photopolymerizable crystalline organic materials and the optimization of the polymerization process becoming the key to further enhancement of the performance of these materials.

## Supplementary Material



















## Notes

Mechanical effects of crystals of **B10** (samples labeled 1–4) of different sizes, and bending and splitting of crystal of **B10** (sample labeled 5) upon exposure to light (MP4)
Phase transition and melting of different samples of **PB10** at 483 K (MP4)
Bending of **B10** and **PB10** crystals at 298 K by applying external force (MP4)
Bending of **B10** and **PB10** crystals at 123 or 77 K (submerged in liquid nitrogen) by applying external force (MP4)
Comparison of the response of **B10** and **PB10** single crystals to pinching with a needle tip (MP4)
Three-point bending of **B10** and **PB10** crystals using a universal mechanical property testing machine (MP4)
Tensile tests of **B10** and **PB10** crystals using a universal mechanical property testing machine (MP4)
Comparison of the load-bearing capacity of **B10** and **PB10** crystals (MP4)
Experimental procedures, ^1^H NMR and ^13^C NMR spectra, absorption and fluorescence emission spectra, SEM and optical images, UV–vis diffuse reflectance spectra, photoinduced curling, photopolymerization, PXRD patterns, crystal growth morphologies, frontier orbital analysis, crystal data and structure refinement, comparison of dimensions and performance metrics, and load-lifting performance (PDF)

## References

[1] Liu Y., He K., Chen G., Leow W. R., Chen X. (2017). Nature-Inspired Structural
Materials for Flexible Electronic Devices. Chem.
Rev..

[2] Root S. E., Savagatrup S., Printz A. D., Rodriquez D., Lipomi D. J. (2017). Mechanical Properties of Organic Semiconductors for
Stretchable, Highly Flexible, and Mechanically Robust Electronics. Chem. Rev..

[3] Li S., Bai H., Shepherd R. F., Zhao H. (2019). Bio-Inspired Design and Additive
Manufacturing of Soft Materials, Machines, Robots, and Haptic Interfaces. Angew. Chem., Int. Ed..

[4] Sun J. Y., Zhao X., Illeperuma W. R. K., Chaudhuri O., Oh K. H., Mooney D. J., Vlassak J. J., Suo Z. (2012). Highly Stretchable
and Tough Hydrogels. Nature.

[5] Kim D.-H., Lu N., Ma R., Kim Y.-S., Kim R.-H., Wang S., Wu J., Won S. M., Tao H., Islam A., Yu K. J., Kim T.-I., Chowdhury R., Ying M., Xu L., Li M., Chung H.-J., Keum H., McCormick M., Liu P., Zhang Y.-W., Omenetto F. G., Huang Y., Coleman T., Rogers J. A. (2011). Epidermal Electronics. Science.

[6] Liao X., Dulle M., De Souza E., Silva J. M., Wehrspohn R. B., Agarwal S., Förster S., Hou H., Smith P., Greiner A. (2019). High Strength in Combination with
High Toughness in
Robust and Sustainable Polymeric Materials. Science.

[7] Ritchie R. O. (2011). The Conflicts
between Strength and Toughness. Nat. Mater..

[8] Xu J., Shao M., Wang X., Chen T., Li S., Zhang X., Wang T., Zhang Y., Yang Z., Wang Q. (2024). Flexible Cages Enable
Robust Supramolecular Elastomers. Adv. Mater..

[9] Kim T., Zhu L., Al-Kaysi R. O., Bardeen C. J. (2014). Organic Photomechanical Materials. ChemPhysChem.

[10] Li Q., Li Z. (2020). Molecular Packing: Another Key Point for the Performance of Organic
and Polymeric Optoelectronic Materials. Acc.
Chem. Res..

[11] Sato O. (2016). Dynamic Molecular
Crystals with Switchable Physical Properties. Nat. Chem..

[12] Sagara Y., Kato T. (2009). Mechanically Induced
Luminescence Changes in Molecular Assemblies. Nat. Chem..

[13] Caruso M.
M., Davis D. A., Shen Q., Odom S. A., Sottos N. R., White S. R., Moore J. S. (2009). Mechanically-Induced Chemical Changes
in Polymeric Materials. Chem. Rev..

[14] Irie M. (2000). Diarylethenes
for Memories and Switches. Chem. Rev..

[15] Al-Kaysi R.
O., Muller A. M., Bardeen C. J. (2006). Photochemically Driven Shape Changes
of Crystalline Organic Nanorods. J. Am. Chem.
Soc..

[16] Zhu H., Yang H., Ma Y., Lu T., Xu F., Genin G. M., Lin M. (2020). Spatiotemporally Controlled Photoresponsive
Hydrogels: Design and Predictive Modeling from Processing through
Application. Adv. Funct. Mater..

[17] Kloxin C. J., Bowman C. N. (2013). Covalent Adaptable
Networks: Smart, Reconfigurable,
and Responsive Network Systems. Chem. Soc. Rev..

[18] Chatani S., Kloxin C. J., Bowman C. N. (2014). The Power
of Light in Polymer Science:
Photochemical Processes to Manipulate Polymer Formation, Structure,
and Properties. Polym. Chem..

[19] Kobatake S., Takami S., Muto H., Ishikawa T., Irie M. (2007). Rapid and
Reversible Shape Changes of Molecular Crystals on Photoirradiation. Nature.

[20] Awad W. M., Davies D. W., Kitagawa D., Halabi J. M., Al-Handawi M. B., Tahir I., Tong F., Campillo-Alvarado G., Shtukenberg A. G., Alkhidir T., Hagiwara Y., Almehairbi M., Lan L., Hasebe S., Karothu D. P., Mohamed S., Koshima H., Kobatake S., Diao Y., Chandrasekar R., Zhang H., Sun C. C., Bardeen C., Al-Kaysi R. O., Kahr B., Naumov P. (2023). Mechanical Properties and Peculiarities
of Molecular Crystals. Chem. Soc. Rev..

[21] Kitagawa D., Tsujioka H., Tong F., Dong X., Bardeen C. J., Kobatake S. (2018). Control of Photomechanical
Crystal Twisting by Illumination
Direction. J. Am. Chem. Soc..

[22] Zhou B., Yan D. (2021). Recent Advances of
Dynamic Molecular Crystals with Light-Triggered
Macro-Movements. Appl. Phys. Rev..

[23] Li P., Ji C., Liu M., Müllen K., Yin M. (2023). Hydrogen-Bonded Homochiral
Molecular Crystals with Mechanical Elasticity and Photomechanical
Bending. Chem. Mater..

[24] Yu Q., Aguila B., Gao J., Xu P., Chen Q., Yan J., Xing D., Chen Y., Cheng P., Zhang Z., Ma S. (2019). Photomechanical Organic
Crystals as Smart Materials for Advanced
Applications. Chem. Eur. J..

[25] Naumov P., Chizhik S., Panda M. K., Nath N. K., Boldyreva E. (2015). Mechanically
Responsive Molecular Crystals. Chem. Rev..

[26] Abendroth J. M., Bushuyev O. S., Weiss P. S., Barrett C. J. (2015). Controlling Motion
at the Nanoscale: Rise of the Molecular Machines. ACS Nano.

[27] Hasebe S., Hagiwara Y., Asahi T., Koshima H. (2024). Actuation Performance
and Versatility of Photothermally Driven Organic Crystals. Angew. Chem., Int. Ed..

[28] Wang H., Chen P., Wu Z., Zhao J., Sun J., Lu R. (2017). Photothermally Responsive Materials. Angew.
Chem., Int. Ed..

[29] Takanabe A., Tanaka M., Johmoto K., Uekusa H., Mori T., Koshima H., Asahi T. (2016). Optical Activity
and Optical Anisotropy
in Photomechanical Crystals of Chiral Salicylidenephenylethylamines. J. Am. Chem. Soc..

[30] Terao F., Morimoto M., Irie M. (2012). Light-Driven
Molecular-Crystal Actuators:
Rapid and Reversible Bending of Rodlike Mixed Crystals of Diarylethene
Derivatives. Angew. Chem., Int. Ed..

[31] Tong F., Kitagawa D., Bushnak I., Al-Kaysi R. O., Bardeen C. J. (2021). Light-Powered
Autonomous Flagella-Like Motion of Molecular Crystal Microwires. Angew. Chem., Int. Ed..

[32] Uchida E., Azumi R., Norikane Y. (2015). Light-Induced
Crawling of Crystals
on a Glass Surface. Nat. Commun..

[33] Athiyarath V., Sureshan K. M. (2020). Designed Synthesis
of a 1D Polymer in Twist-Stacked
Topology via Single-Crystal-to-Single-Crystal Polymerization. Angew. Chem., Int. Ed..

[34] Rai R., Khazeber R., Sureshan K. M. (2023). Single-Crystal-to-Single-Crystal
Topochemical Synthesis of a Collagen-Inspired Covalent Helical Polymer. Angew. Chem., Int. Ed..

[35] Wang M., Jin Y., Zhang W., Zhao Y. (2023). Single-Crystal Polymers (SCPs): From
1D to 3D Architectures. Chem. Soc. Rev..

[36] Pathan J.
R., Balan H., Das K., Reddy C. M., Sureshan K. M. (2025). Single-Crystal-to-Single-Crystal
Synthesis of a Polymer in Two Distinct Topologies. Angew. Chem., Int. Ed..

[37] Shan T., Chen L., Guo Z., Xiao D., Wang M., Xiao X., Li G., Huang F. (2025). Designing High-Mechanical-Property
Organic Polymeric Crystals: Insights from Stress Dispersion and Energy
Dissipation Strategies. J. Am. Chem. Soc..

[38] Corrigan N., Yeow J., Judzewitsch P., Xu J., Boyer C. (2019). Seeing the
Light: Advancing Materials Chemistry through Photopolymerization. Angew. Chem., Int. Ed..

[39] Cao C., Xue X.-R., Ge Y., Liu D., Braunstein P., Lang J.-P. (2024). Photodimerization-Triggered Photopolymerization
of
Triene Coordination Polymers Enables Macroscopic Photomechanical Movements. J. Am. Chem. Soc..

[40] Bhandary S., Beliš M., Shukla R., Bourda L., Kaczmarek A. M., Van Hecke K. (2024). Single-Crystal-to-Single-Crystal Photosynthesis of
Supramolecular Organoboron Polymers with Dynamic Effects. J. Am. Chem. Soc..

[41] Guo Q.-H., Jia M., Liu Z., Qiu Y., Chen H., Shen D., Zhang X., Tu Q., Ryder M. R., Chen H., Li P., Xu Y., Li P., Chen Z., Shekhawat G. S., Dravid V. P., Snurr R. Q., Philp D., Sue A. C.-H., Farha O. K., Rolandi M., Stoddart J. F. (2020). Single-Crystal Polycationic
Polymers Obtained by Single-Crystal-to-Single-Crystal Photopolymerization. J. Am. Chem. Soc..

[42] Li M., Schlüter A. D., Sakamoto J. (2012). Solid-State Photopolymerization of
a Shape-Persistent Macrocycle with Two 1,8-Diazaanthracene Units in
a Single Crystal. J. Am. Chem. Soc..

[43] Dou L., Zheng Y., Shen X., Wu G., Fields K., Hsu W., Zhou H., Yang Y., Wudl F. (2014). Single-Crystal Linear
Polymers through Visible Light–Triggered Topochemical Quantitative
Polymerization. Science.

[44] Wei Z., Wang X., Seo B., Luo X., Hu Q., Jones J., Zeller M., Wang K., Savoie B. M., Zhao K., Dou L. (2022). Side-Chain Control
of Topochemical
Polymer Single Crystals with Tunable Elastic Modulus. Angew. Chem., Int. Ed..

[45] Hema K., Ravi A., Raju C., Sureshan K. M. (2021). Polymers with Advanced
Structural and Supramolecular Features Synthesized through Topochemical
Polymerization. Chem. Sci..

[46] Rath B. B., Vittal J. J. (2022). Photoreactive Crystals
Exhibiting [2 + 2] Photocycloaddition
Reaction and Dynamic Effects. Acc. Chem. Res..

[47] Jiang Z., Zhao H., Wu W., Chen K., Yu H., Wang T., Huang X., Wang N., Zhou L., Hao H. (2023). Multi-stimuli Responsive Organic Polymorphic Crystals: Anisotropic
Elasticity and Plasticity, Mechanochromism and Photomechanical Motions. J. Mater. Chem. C.

[48] Wang Y., Chen Y., Wang F., Lu S., Chen X. (2025). Photoresponsive
Single Crystals of Organic Small Molecules. Dyes Pigm..

[49] Long L., Rivero S. M., Sun F., Wang D., Chekulaev D., Tonnelé C., Casanova D., Casado J., Zheng Y. (2023). A Single-Crystal
Monomer to Single-Crystal Polymer Reaction Activated by a Triplet
Excimer in a Zipper Mechanism. Angew. Chem.,
Int. Ed..

[50] Timoshenko, S. History of Strength of Materials; McGraw-Hill: New York, 1953.

[51] Shan T., Chen L., Guo Z., Xiao D., Wang M., Xiao X., Li G., Huang F. (2025). Designing
High-Mechanical-Property
Organic Polymeric Crystals: Insights from Stress Dispersion and Energy
Dissipation Strategies. J. Am. Chem. Soc..

[52] Mazzotta M. G., Putnam A. A., North M. A., Wilker J. J. (2020). Weak Bonds in a
Biomimetic Adhesive Enhance Toughness and Performance. J. Am. Chem. Soc..

