# Supplementary material for: Stiffening Organic Crystals through Polymerization Using Visible Light
Source: J Am Chem Soc. 2025 Oct 25;147(44):41034–45. doi: 10.1021/jacs.5c15122 (PMC12593339; doi:10.1021/jacs.5c15122)
Supplement: Supplementary file 9 [file ja5c15122_si_009.pdf]

Supporting Information

Stiffening Organic Crystals through Polymerization Using Visible Light

Linfeng Lan,[1,2] Yuxing Zhou,[1] Liang Li,[3,4] Chenguang Wang,[2] Pance Naumov[3,5,6,7,*], Hongyu Zhang[1,*]

[1]State Key Laboratory of Supramolecular Structure and Materials, College of Chemistry, Jilin University, Changchun, 130012, P. R. China
[2]State Key Laboratory of Integrated Optoelectronics, College of Electronic Science and Engineering, Jilin University, Changchun, 130012, P. R. China
[3]Smart Materials Lab, New York University Abu Dhabi, PO Box 129188, Abu Dhabi, UAE
[4]Department of Sciences and Engineering Department, Sorbonne University Abu Dhabi, PO Box 38044, Abu Dhabi, UAE
[5]Center for Smart Engineering Materials, New York University Abu Dhabi, PO Box 129188, Abu Dhabi, UAE
[6]Research Center for Environment and Materials, Macedonian Academy of Sciences and Arts, Bul. Krste Misirkov 2, Skopje, MK–1000, Macedonia
[7]Molecular Design Institute, Department of Chemistry, New York University, 100 Washington Square East, New York, NY 10003, USA

*Corresponding authors. Emails for correspondence: pance.naumov@nyu.edu; hongyuzhang@jlu.edu.cn

The PDF file includes:

Materials and Methods
Figs. S1 to S47
Tables S1 to S8
Legends for the supplementary movies
References

Other Supplementary Materials for this manuscript include the following:

Movies S1 to S8

Table of Contents

1. Supplementary methods **3**

2. Supplementary figures **7**

3. Supplementary tables **33**

4. Legends for the supplementary movies **39**

5. Supplementary references **40**

1. Supplementary methods

Preparation. The materials for organic syntheses were obtained from commercial sources and were used as received. The compound 1,1,′-dioxo-1*H*,1′*H*-[2,2′-biindene]-3,3′-diylbis(decanoate) (**B10**) was synthesized according to the following procedure (Scheme S1):

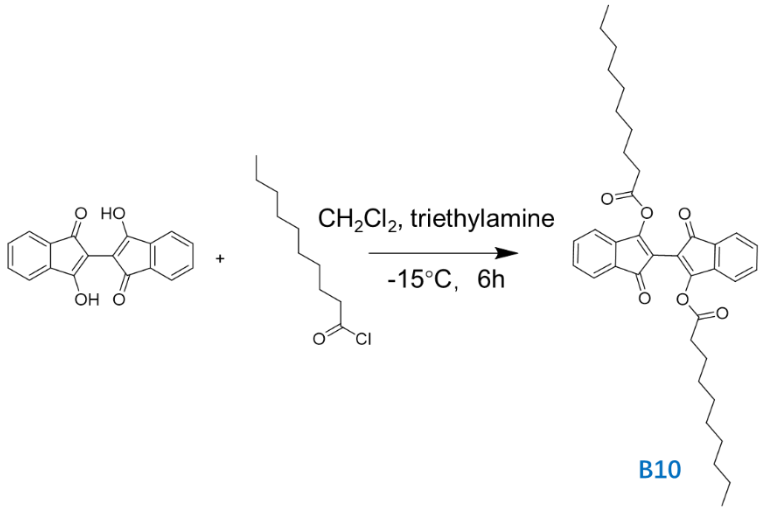

Scheme S1. Synthesis of **B10**.

First, [2,2′-bi-1*H*-indene]-3,3′- dihydroxy-1,1′-dione was synthesized according to the methods reported in the literature.[1,2] Subsequently, 1.45 g (5.0 mmol) of this compound was added to a dry two-neck round-bottom flask, followed by addition of 50 mL anhydrous dichloromethane under nitrogen. The mixture was cooled to −15 °C in an ice-salt bath, and 2.8 mL (20 mmol) triethylamine was added. Decanoyl chloride (4.1 mL, 20 mmol) was then dripped into the solution over a period of 15 minutes. The reaction was allowed to proceed at −15°C for 6 hours, whereupon orange-red solids precipitated out. The reaction was quenched with water, and after washing with saturated saline solution and drying with anhydrous sodium sulfate, the crude product was obtained by roto-evaporation. Column chromatography was performed on the crude product by elution with a mixture of dichloromethane and petroleum ether in a volume ratio of 1:2. Recrystallization was carried out by adding 5 mL of cold methanol to the flask, and filtration yielded **B10** as an orange powder (1.56 g, yield: 52.1%). ^{1}H NMR (400 MHz, chloroform-*d*) δ 7.43 (d, *J* = 7.1 Hz, 2H), 7.40 – 7.34 (m, 2H), 7.30 – 7.27 (m, 2H), 7.10 (d, *J* = 7.2 Hz, 2H), 2.65 (t, *J* = 7.4 Hz, 4H), 1.72 (p, *J* = 7.5 Hz, 4H), 1.26 (s, 24H), 0.88 (t, *J* = 6.8 Hz, 6H). ^{13}C NMR (101 MHz, chloroform-*d*) δ 192.40, 169.25, 164.43, 140.41, 133.40, 130.89, 129.93, 122.34, 119.93, 112.62, 34.23, 31.92, 29.47, 29.32, 29.05, 24.52, 22.72, 14.16, 1.07.

Crystal growth. 1 g (1.67 mmol) of **B10** was dissolved in 10 mL of dichloromethane in a 50 mL flask. Subsequently, 20 mL of ethanol was slowly added along the inner wall of the flask. The solution was left undisturbed in the dark at 278 K for about one week, resulting in the formation of a large number of needle-like **B10** crystals. A portion of the **B10** crystals was exposed to white light (2.5 W/cm^2) until they changed their color to white. The crystals were then washed with dichloromethane and ethanol to remove any unreacted **B10** and dried in air to obtain crystals of **PB10**. To minimize photodegradation, crystals of **B10** were first encapsulated in polyvinyl alcohol (PVA, M.W. 24,500, alcoholysis degree: 86.5–89.0 mol%). The encapsulated crystals were gradually converted to **PB10** under natural light. Finally, the PVA coating on the crystal surface was removed by washing with hot water, resulting in the formation of large-sized **PB10** crystals. Based on the difference in solubility of **B10** and **PB10** in dichloromethane (the saturated solution concentration of the former is ~0.2 g/mL, while the latter is completely insoluble), we repeatedly rinsed the **B10** crystals after full photopolymerization with dichloromethane five times and naturally dried them to obtain polymer single crystals without monomer components for all subsequent tests.

X-ray single crystal diffraction. Diffraction data of **B10** and **PB10** at 298 K and 100 K were collected on a Bruker D8 Venture diffractometer. The data collection, integration, scaling, and absorption corrections were performed using the Bruker Apex 3 software.[3] The structures were solved with direct methods using the Olex2 program suite and refined with full-matrix least-squares on F^2.[4] Non-hydrogen atoms were refined anisotropically. The positions of the hydrogen atoms were calculated and refined isotropically. The graphics related to the structures were generated by using Mercury 4.2.0.[5] The crystallographic information has been deposited at the Cambridge Crystallographic Data Centre (CCDC) with the following CCDC numbers: 2412992 for **B10** at 100 K, 2412993 for **PB10** at 298 K and 2412994 for **PB10** at 100 K.

Characterization. ^{1}H and ^{13}C{^{1}H} NMR spectra were recorded on an Agar Scientifica 400 MHz spectrometer (^{1}H: 400 MHz; ^{13}C{^{1}H}: 101 MHz) with tetramethylsilane as the internal standard. The UV−vis absorption spectra were recorded with a Shimadzu UV-2550 spectrophotometer. The emission spectra were recorded with the Maya2000 Pro CCD spectrometer. Optical photographs of crystals were obtained by using a Canon camera, an optical Olympus BX61 microscope and a DSX1000 3D digital ultra depth of field microscope. Scanning electron microscopy (SEM) images were obtained on a Regulus8100 field emission transmission electron microscope operated at 3–5 kV. Powder X-ray diffraction data were collected on a SmartLab (3) X-ray diffractometer. Differential scanning calorimetric (DSC) measurements were carried out on a Dsc-3/500 differential scanning calorimeter.

The three-point bending and tensile tests were performed using an Instron 5944 universal testing system with a capacity of 5 N or 10 N Instron 2530 load cell. The surface texture and roughness were analyzed with a Bruker ICON-XR atomic force microscope (AFM). The nanoindentation measurements were conducted with Agilent Nano Indenter G200 with the CSM method and an XP-style actuator.

Measurement of the extension/contraction ratio (*ε*). When the crystal is bent to its limit, the extension/contraction ratio (*ε*) of the outer/inner arc can be estimated by the equation[3,4]:

$$\text{Inner arc} = \frac{1}{2} \times \pi \times d$$

$$\text{Outer arc} = \frac{1}{2} \times \pi \times (d + 2t)$$

$$\text{Initial length} \approx \text{middle arc length} = \frac{1}{2} \times \pi \times (d + t)$$

Therefore,

$$\varepsilon = \frac{\text{Outer arc length-Initial length}}{\text{Initial length}} \text{ or } \frac{\text{Initial length-Inner arc length}}{\text{Initial length}} = \frac{t}{d+t} \times 100\ (\%)$$

where t is the thickness of the crystal and d is the diameter of the inner arc, which is obtained by measuring the radius of the curvature when the crystal is bent to its maximum degree.

Computational methods and analysis. The crystal growth morphologies were simulated using the Bravais-Friedel-Donnay-Harker (BFDH) method with Materials Studio 2019.[5] The HOMO, LUMO of **B10** were calculated at the B3LYP/6-31G (d,p) level by using the Gaussian 09 software, with Grimme's D3 version of dispersion correction applied.[6] Interaction energies were calculated at B3LYP/6-31G(d,p) level of theory using Crystal Explorer 17.5.[7] Energy frameworks and different types of interaction energies along different directions for **B10** are shown in Figure S24. Key intermolecular interactions present in the crystal structures of **B10** and **PB10** were analyzed using Hirshfeld surface analysis in Crystal Explorer 17.5.[7] Due to the computational limitations in handling excessively long polymer chains, we selected trimer and decamer segments from the PB10 crystal structure for Hirshfeld surface analysis. The Hirshfeld surface is defined as a set of points in the 3D space where the ratio of promolecule and procrystal electron densities equals 0.5.[8] Intermolecular interactions were examined by mapping normalized contact distances (d_{norm}), which is a function of the closest distance from the point to the nuclei interior (d_{i}) and exterior (d_{e}) to the surface as well as on the van der Waals radii (r_{vdW}). The proportions of different interactions for **B10** and **PB10** identified through this analysis are illustrated using the fingerprint plots in Figures S34 to S36.

Three-point bending experiments. The three-point bending test was conducted on an Instron 5944 universal testing machine equipped with a 5 N sensor and R3 three-point bending test accessories. The crystal was placed centrally on a support frame with a fixed span, and a vertical downward load was applied at the midpoint between the two support points (crosshead speed: 2 mm/min). The three contact points formed equal moments, resulting in three-point bending, and the corresponding load-displacement curve was recorded. The crystal dimensions were measured using an optical microscope and were used to calculate corresponding stress-strain curve and elastic modulus.[12]

Tensile experiments. The tensile test was conducted using an Instron 5944 universal testing machine equipped with a 10 N sensor and tensile test fixtures. The two ends of the crystal were vertically fixed to the upper and lower grips using UV-curing adhesive or 502 Super Glue, ensuring the crystal was perfectly aligned and parallel to the grips. The crosshead speed was set to 1 mm/min. The initial length of the crystal was measured with a Vernier caliper, while the width and thickness were determined using an optical microscope. These measurements were used to calculate the stress-strain curve and the tensile modulus.[12]

2. Supplementary figures

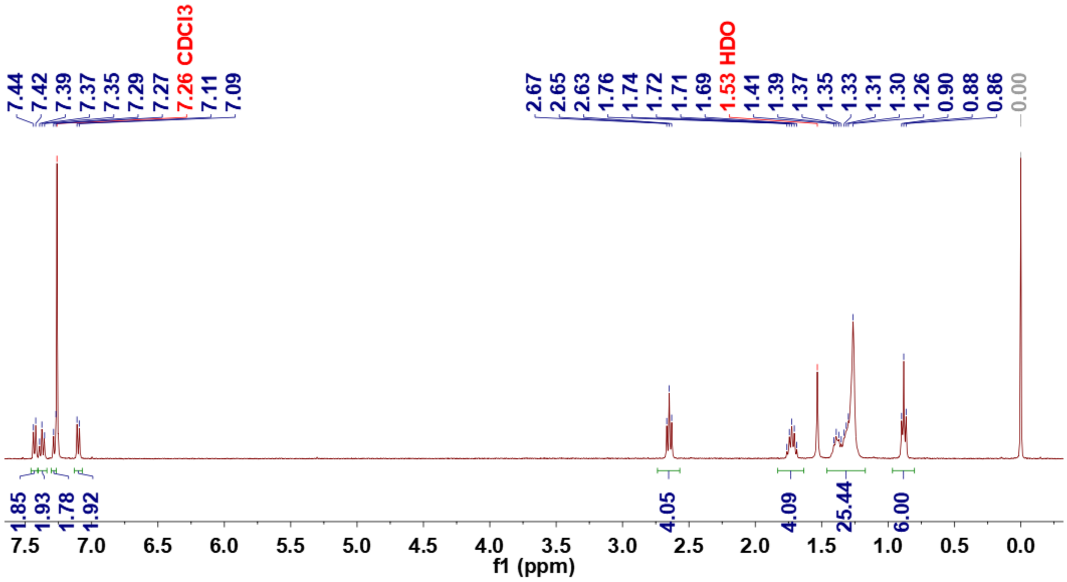

Figure S1. ^{1}H NMR spectrum of **B10** in $CDCl_3$ (400 MHz).

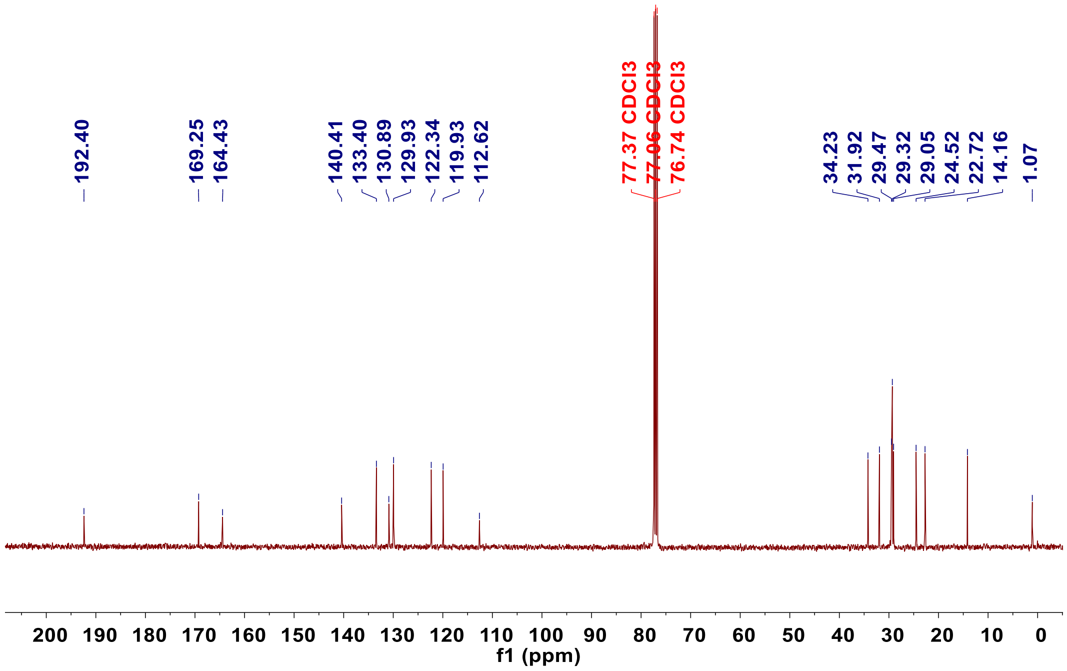

Figure S2. ^{13}C NMR spectrum of **B10** in $CDCl_3$ (101 MHz).

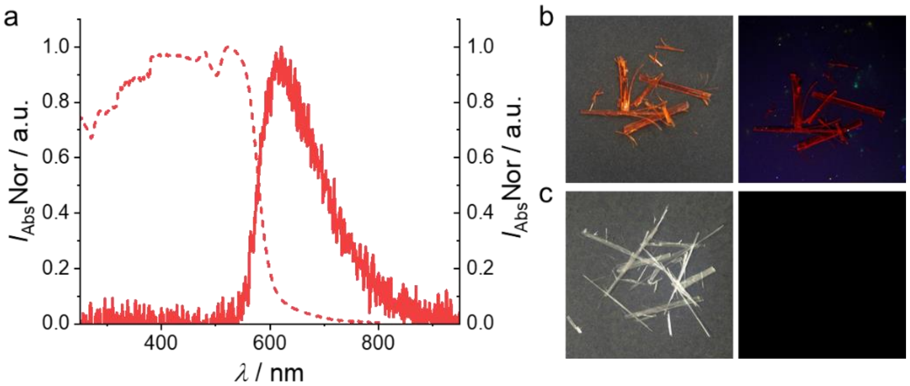

Figure S3. (a) Absorption (broken line) and fluorescence emission spectra (solid line) of **B10** recorded from single crystals. (b,c) Photographs of **B10** and **PB10** crystal recorded in daylight (left) and under UV light (right).

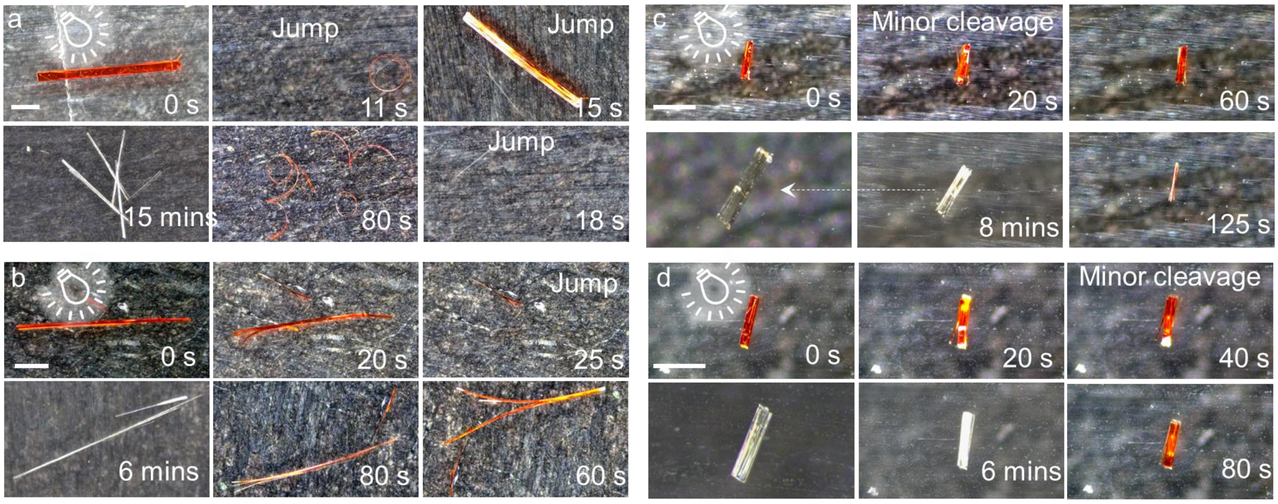

Figure S4. Sequential optical images showing the jumping motion of 1 cm crystals (a,b) under white light irradiation (2.5 W/cm^2) at different time points, and the splitting processes of 2 mm crystals, and (c,d) under prolonged white light exposure.

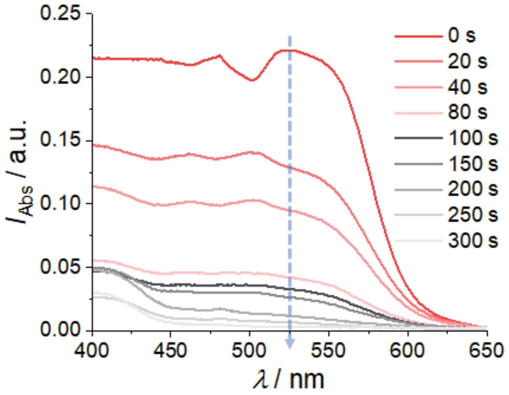

Figure S5. Change in solid-state UV–Vis diffuse reflectance spectra (Kubelka–Munk transformed) during the crystalline-state polymerization of **B10** under white light irradiation (2.5 W/cm^2) at room temperature. The blue arrow represents the change of signature absorption peak around 525 nm.

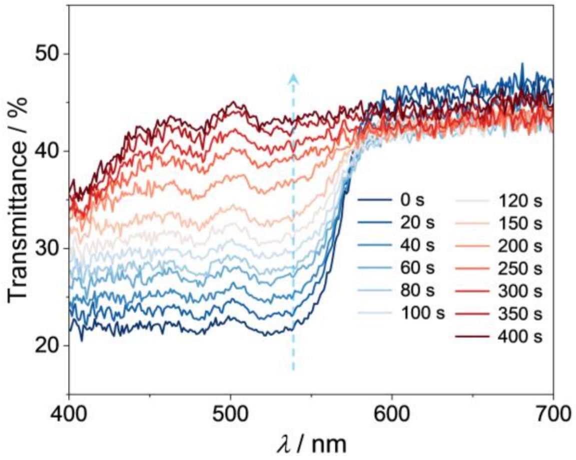

Figure S6. Change in solid-state transmission spectra during the crystalline-state polymerization of **B10** under white light irradiation (2.5 W/cm^2) at room temperature.

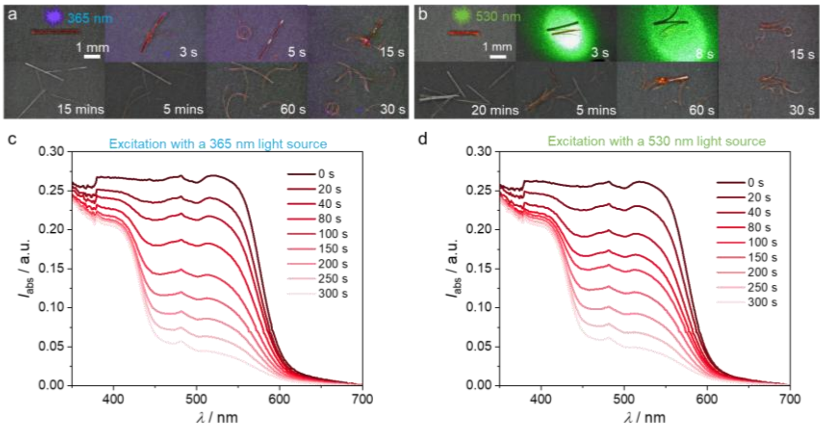

Figure S7. (a,b) Time-resolved optical images of the single-crystal-to-single-crystal transformation of **B10** irradiation with 365 nm (a) and 530 nm light source (b). (c,d) Change in solid-state UV–Vis diffuse reflectance spectra during the crystalline-state polymerization of **B10** irradiation with 365 nm (c) and 530 nm light source (d).

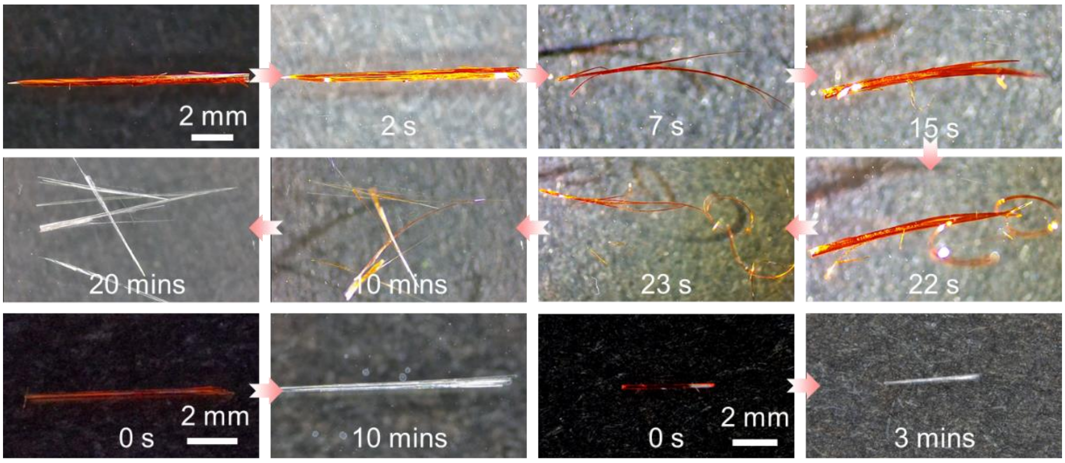

Figure S8. The transformation of **B10** crystals under the same light irradiation demonstrates the effect of size. Larger crystal sizes result in more efficient photopolymerization processes and require longer irradiation times.

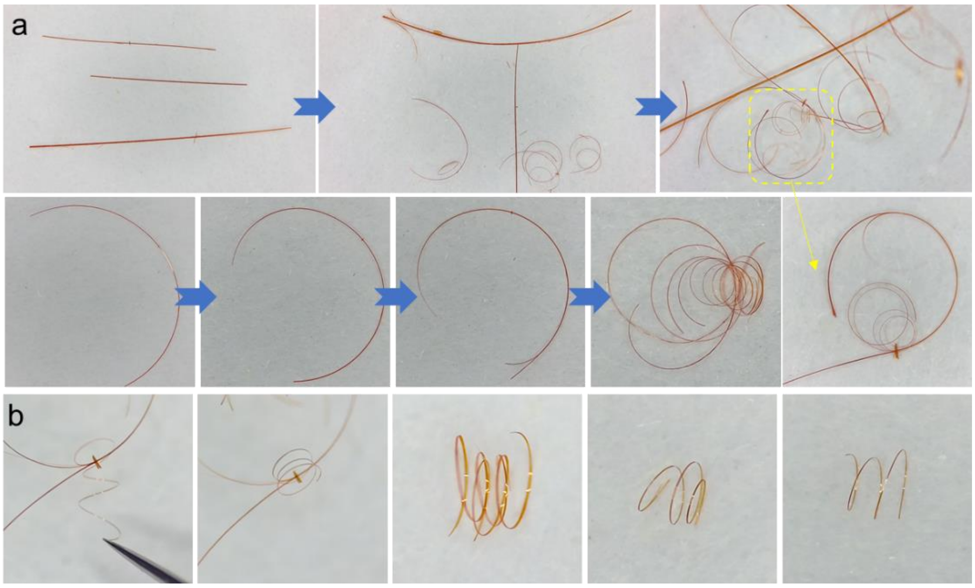

Figure S9. (a,b) The process of photoinduced curling of several **B10** crystals into a mass of spirals (a), and the resulting elastic three-dimensional (3D) spring-like crystals (b).

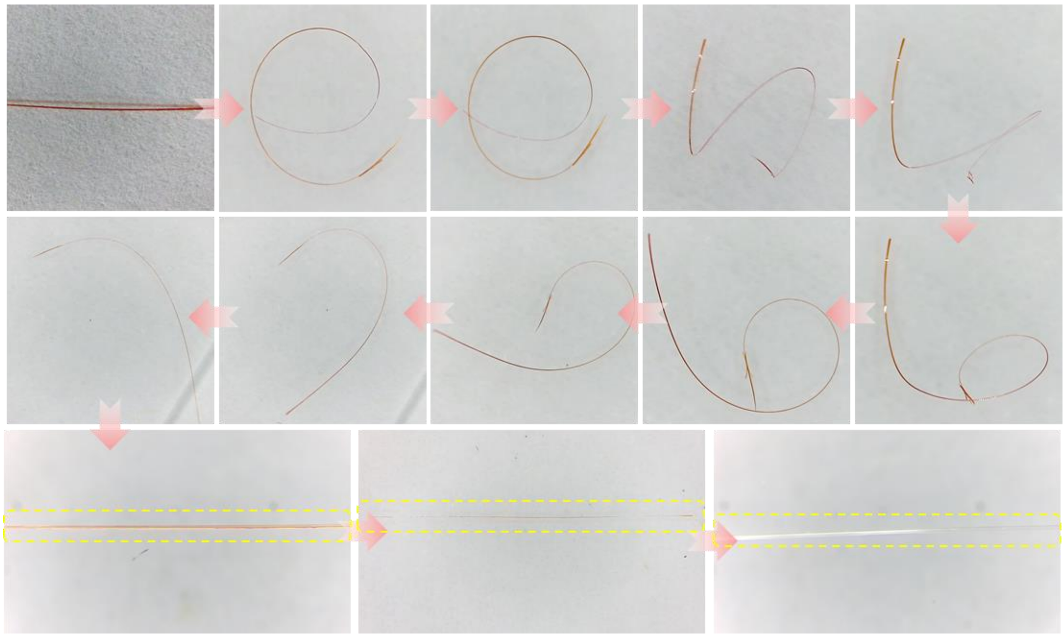

Figure S10. The transformation of a long **B10** single crystal (approximately 2 cm in length, 34.11 μm in width, and 8.68 μm in thickness) into a **PB10** single crystal occurs without splitting.

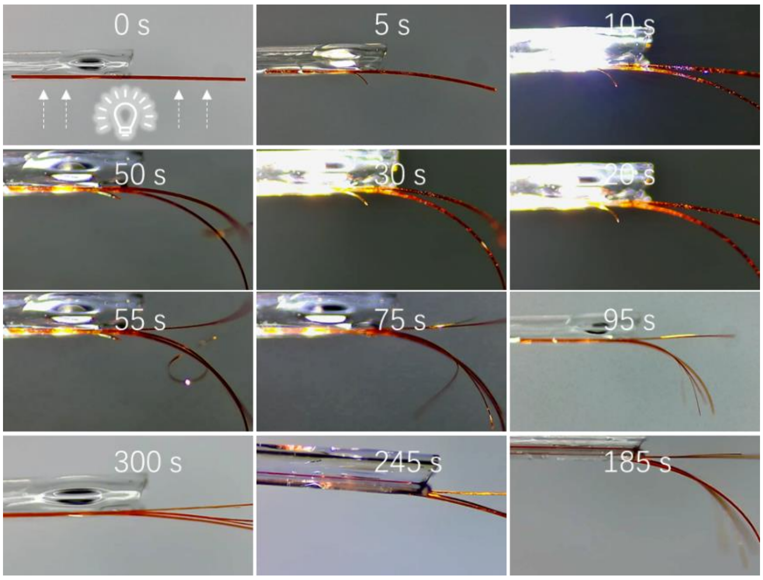

Figure S11. Bending, curling, and straightening process of the crystal toward the light source (supplement to Figure 2e).

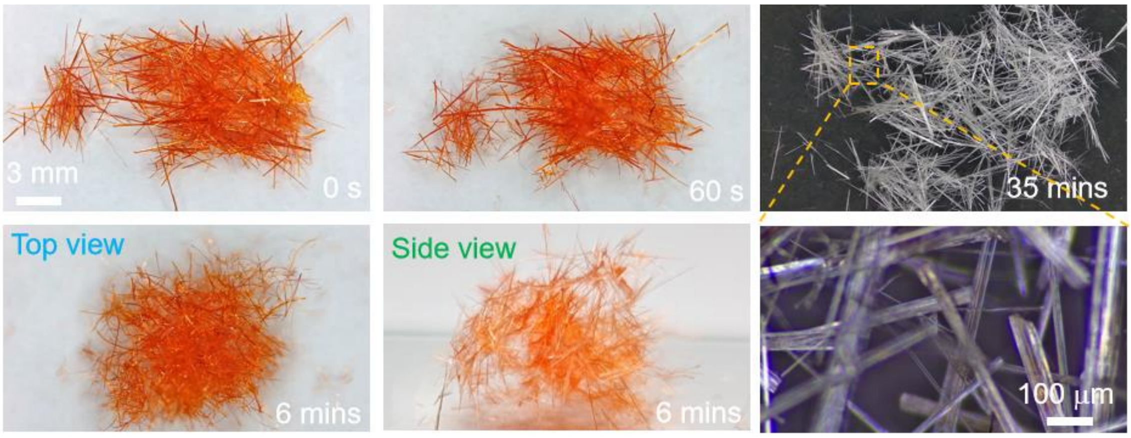

Figure S12. Photopolymerization of a collection of **B10** crystals. Images show that at 0 s the crystals are straight and bright orange in color. Top-view and side-view images at 6 minutes show that the crystals become entangled, forming a two-dimensional network-like structure while gradually fading in color. After 60 s of irradiation, the crystals begin to split and curl. By 35 minutes, the crystals become fully bleached and straighten again. The high-magnification optical image (bottom right) highlights that the crystals remain transparent and structurally intact after photopolymerization.

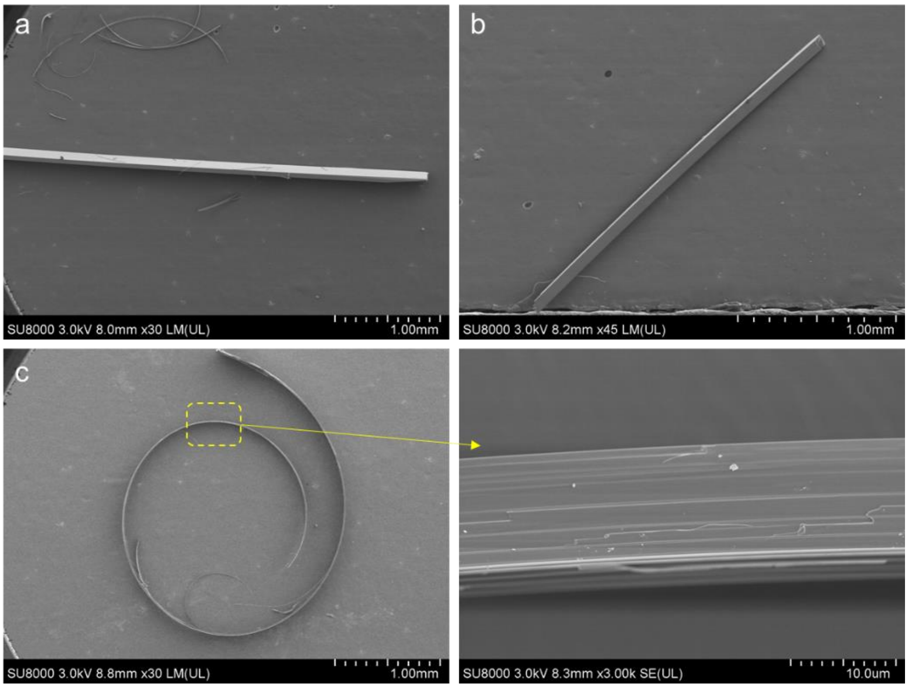

Figure S13. (a–c) Scanning electron microscopy (SEM) images of the surface morphology of **B10** (a) and **PB10** (b) crystals, and crystals in a curled state obtained by photoreaction (c).

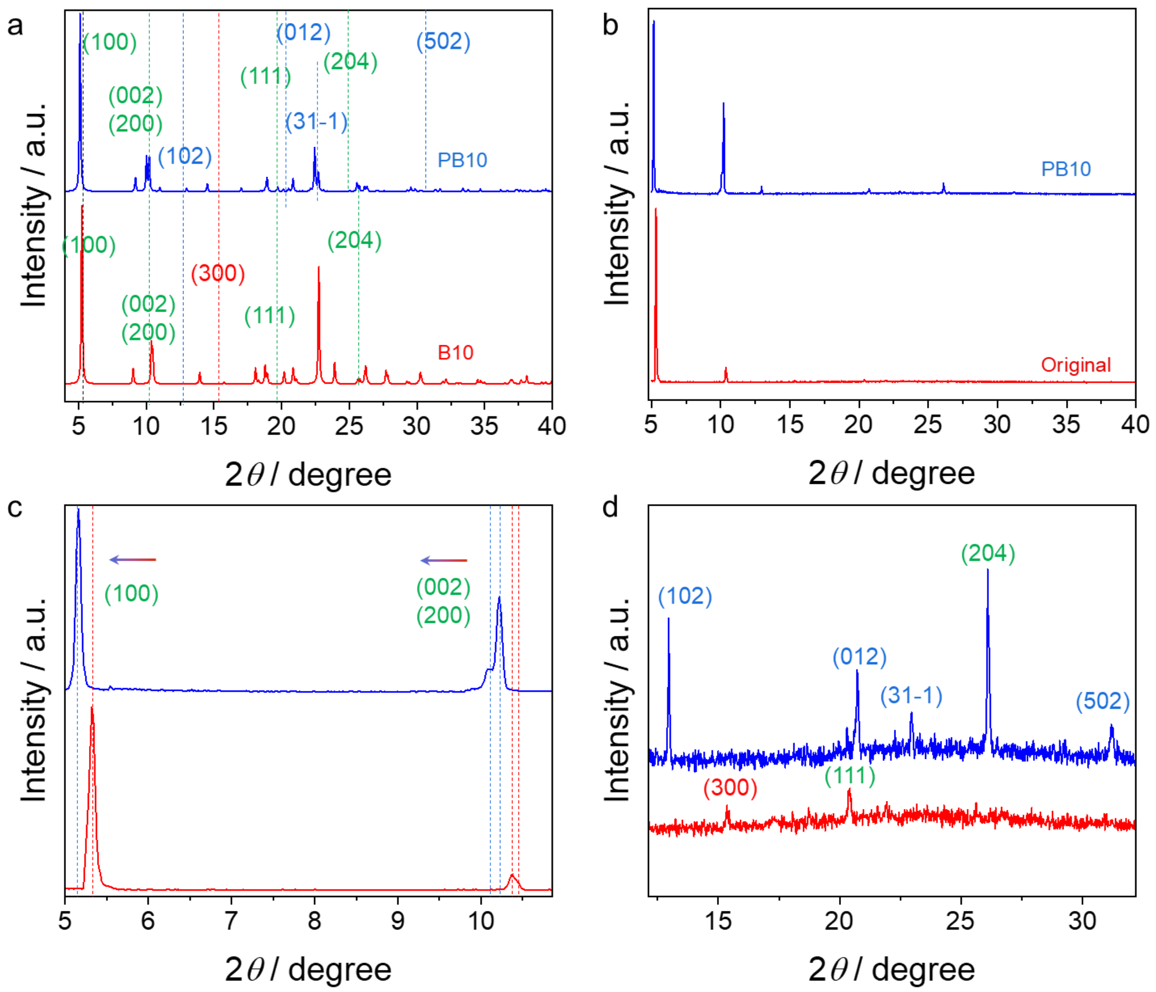

Figure S14. (a,b) PXRD patterns of **B10** and **PB10** crystals obtained through single-crystal simulation (a) and experimental testing (b). (c,d) Magnified view of the PXRD patterns showing changes in characteristic peaks. The Miller indices are color-labeled: green for common peaks for **B10** and **PB10**, red for **B10** peaks, and blue for **PB10** peaks.

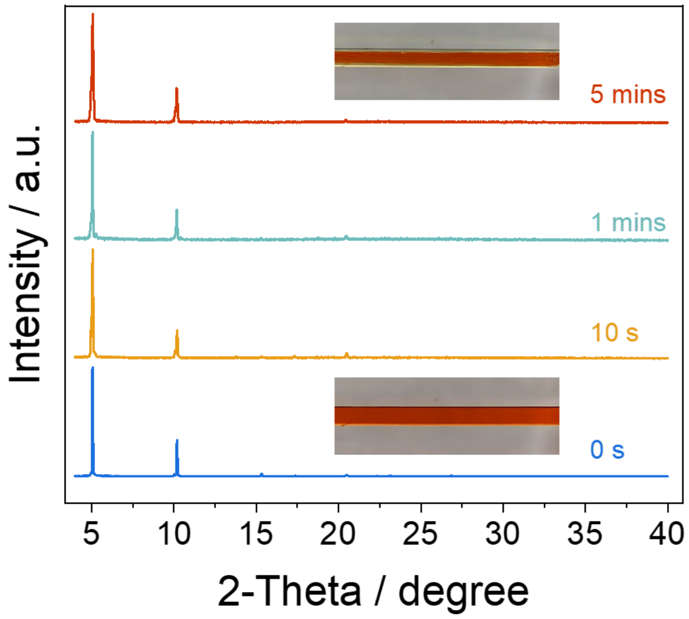

Figure S15. Wide-range angle XRD patterns of the *in situ* photopolymerization process of **PB10** crystals over 5 minutes (corresponding to Figure 2g). The inset shows *in situ* photopolymerization of **B10** crystals encapsulated in polyvinyl alcohol (PVA).

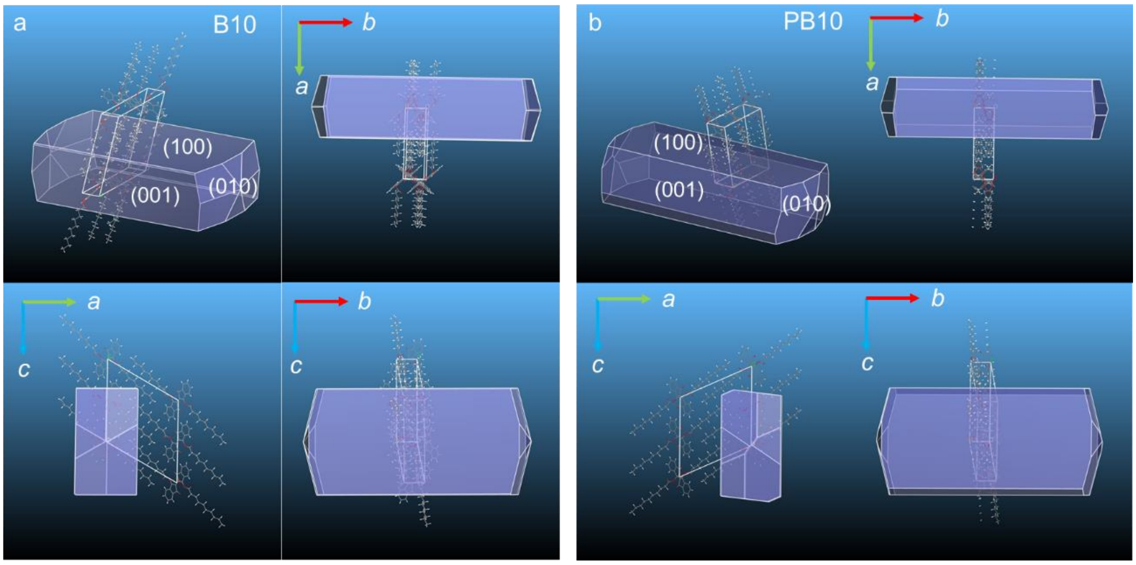

Figure S16. Crystal growth morphologies of **B10** (a) and **PB10** (b) modelled by the software Materials Studio2019.

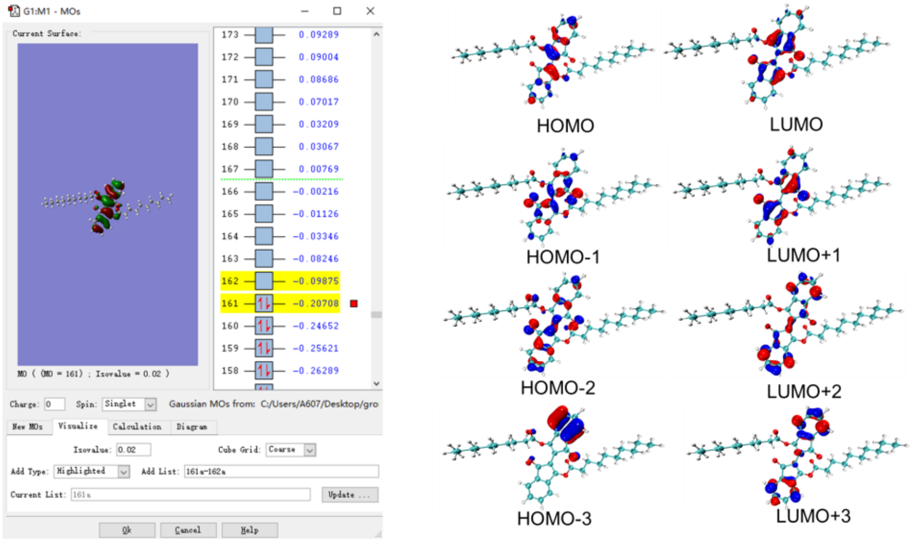

Figure S17. Frontier orbital analysis of the **B10** crystal.

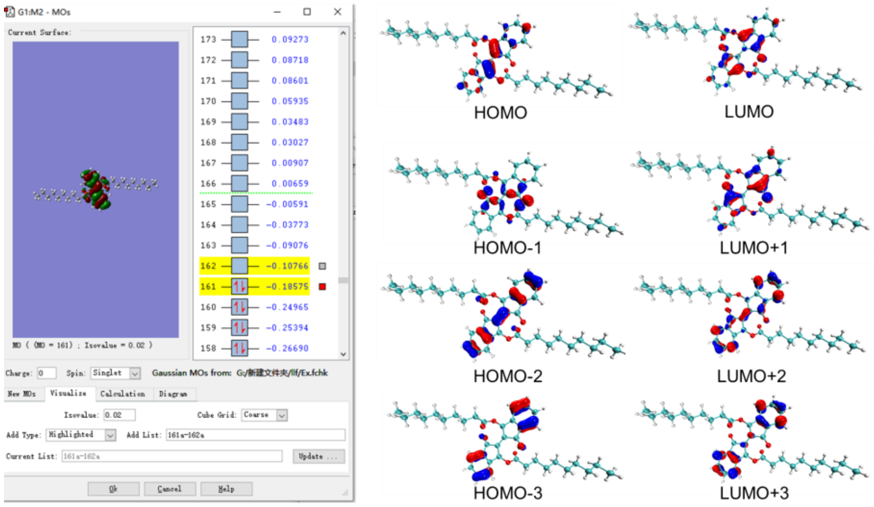

Figure S18. Frontier orbital analysis of the **PB10** crystal.

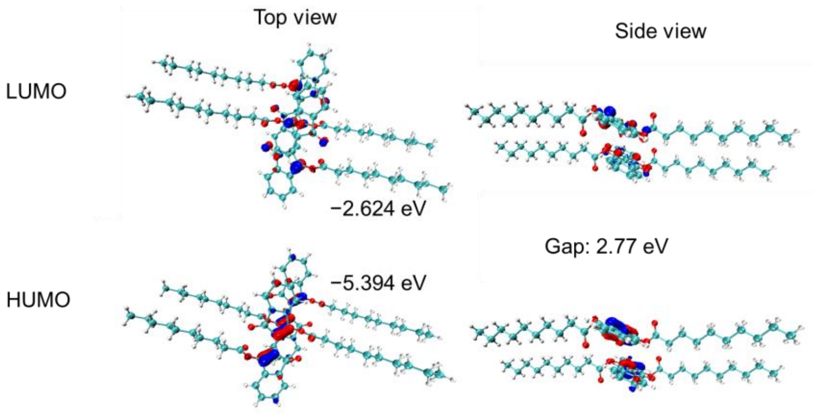

Figure S19. Frontier molecular orbital analysis of **B10** dimer based on single-crystal structure.

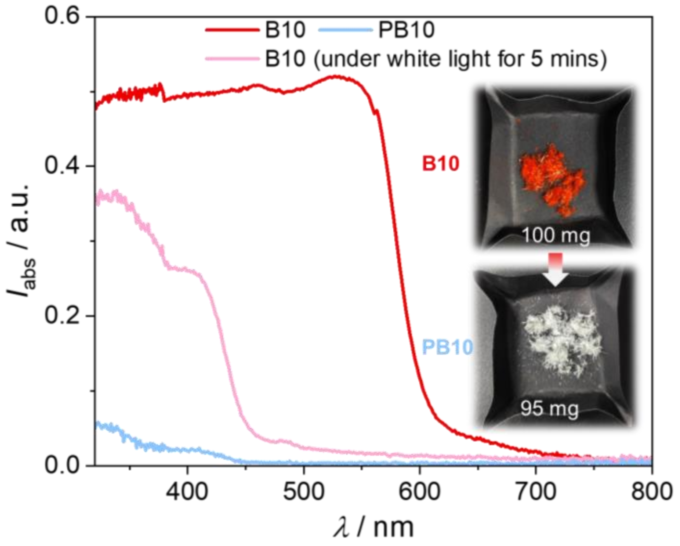

Figure S20. Solid-state UV–Vis diffuse reflectance spectra of **B10** crystals before irradiation, after 5 minutes of light exposure, and of sufficiently polymerized **PB10** crystals (after 2 hours of irradiation). Barium sulfate ($BaSO_4$) was used as the non-absorbing reference. The inset shows photographs of crystals before and after photopolymerization. To ensure the absence of residual monomer, the polymerized crystals were repeatedly washed with dichloromethane and subsequently weighed.

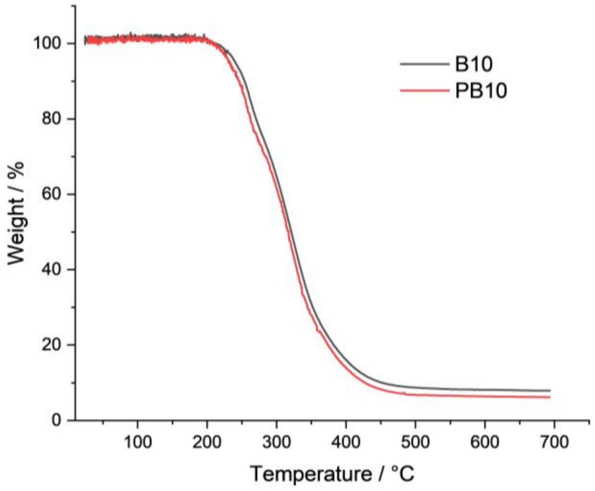

Figure S21. TGA curves of **B10** monomer crystals and **PB10** polymerized crystals under a nitrogen atmosphere.

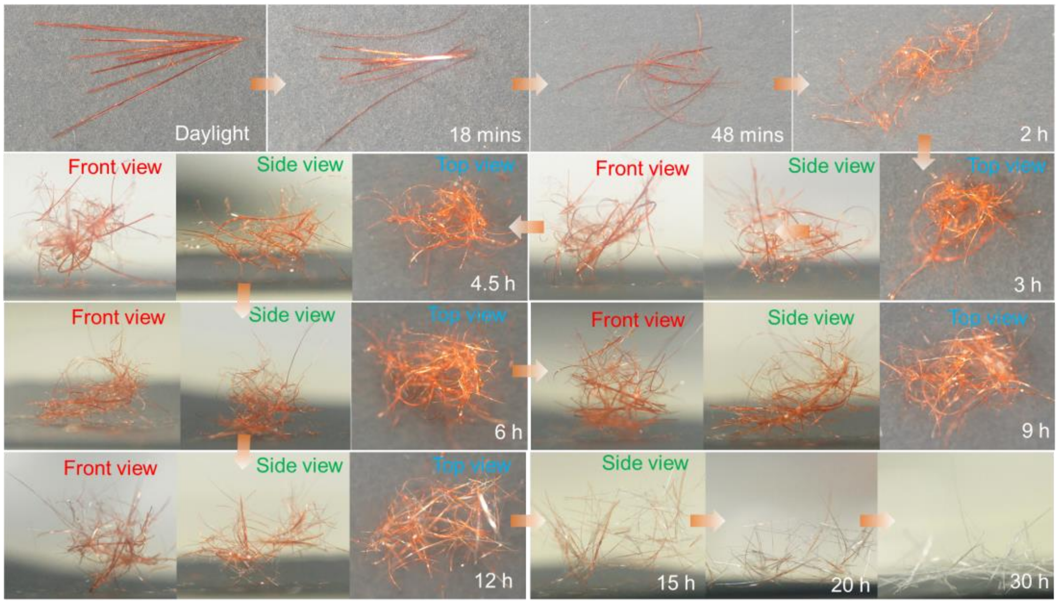

Figure S22. Photoreaction process of several **B10** crystals under natural light, showing the gradual bending, splitting, additional splitting, and entangling into a three-dimensional network of crystals, followed by whitening and straightening as the crystal is converted completely.

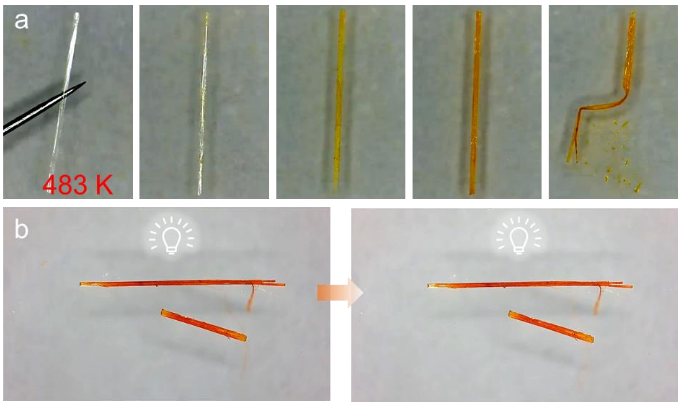

Figure S23. (a) The gradual conversion of **PB10** into the red monomer at approximately 483 K, followed by rapid melting. (b) The resulting monomer after the phase transition that cannot undergo photopolymerization.

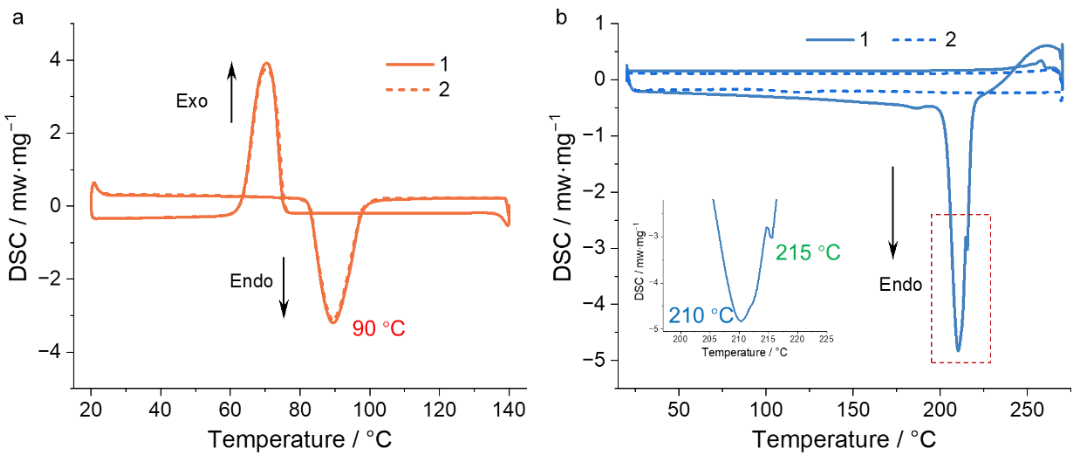

Figure S24. (a) Two heating-cooling cycles in the differential scanning calorimetry (DSC) analysis of **B10**. (b) DSC curves of **PB10** with local amplification. The absence of crystallization peaks in the first cycle and the absence of peaks in the second cycle indicate that the resulting monomer is amorphous.

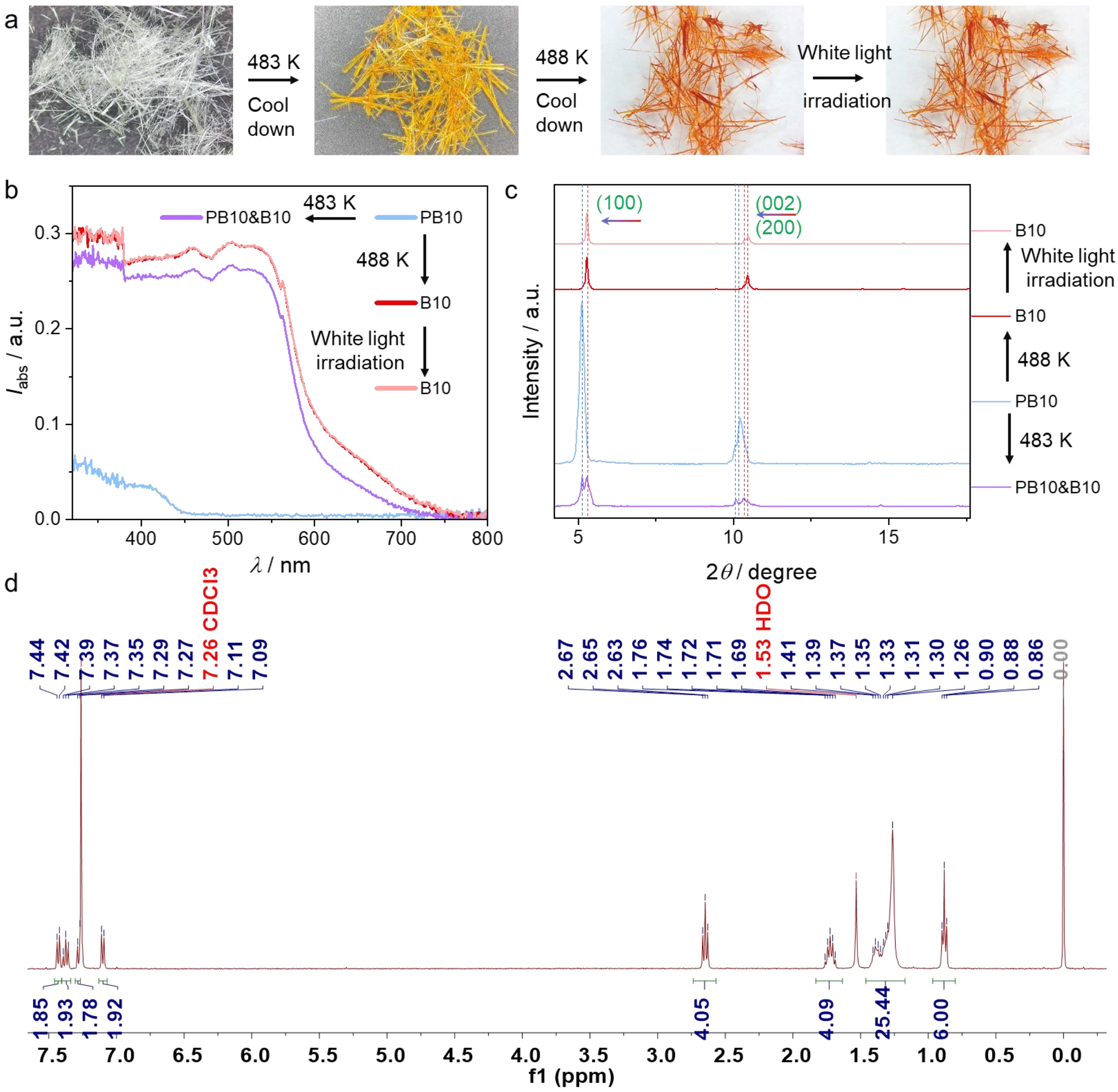

Figure S25. (a) Morphological changes of **PB10**: initial **PB10** crystals, depolymerization products obtained by natural cooling after heating to 483 K and 488 K, and depolymerization products under white light irradiation. (b,c) Corresponding UV−Vis absorption diffuse reflectance spectra (b) and PXRD patterns (c). (d) ^{1}H NMR spectrum of the depolymerization products in $CDCl_3$.

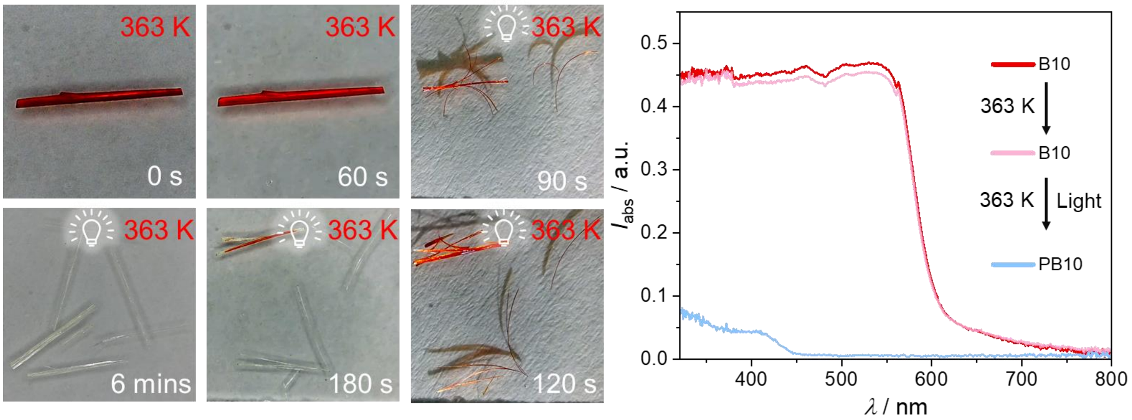

Figure S26. Photographs of a single crystal **B10** under high temperature without light and during photoreaction under light exposure and the corresponding UV–Vis absorption spectra in the crystalline state using diffuse reflectance mode. Barium sulfate ($BaSO_4$) was used as the non-absorbing reference.

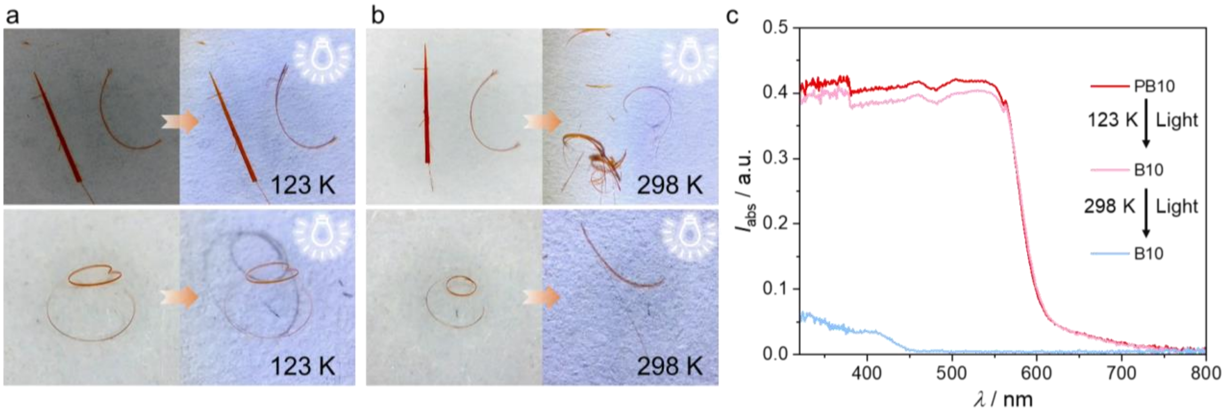

Figure S27. (a,b) Photographs of straight, bent, and helical crystals under white light irradiation at low temperature (a) and room temperature (b). (c) UV–Vis absorption spectra were recorded for **PB10** in the crystalline state using diffuse reflectance mode. Barium sulfate ($BaSO_4$) was used as the non-absorbing reference.

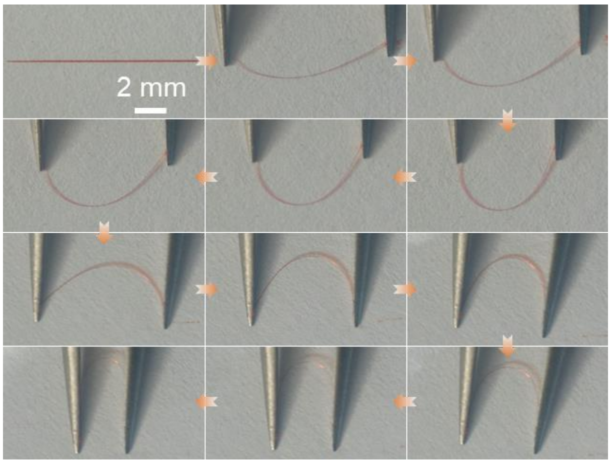

Figure S28. The detailed process of a **B10** crystal being repeatedly bent with tweezers at 298 K, as a supplement to Figure 4b.

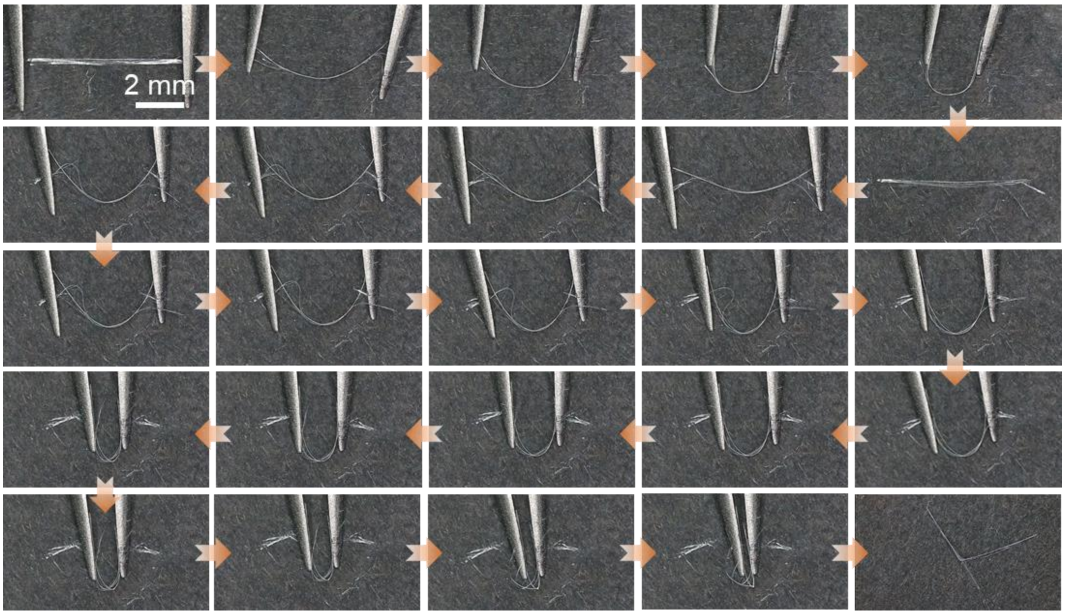

Figure S29. The detailed process of a **PB10** crystal being repeatedly bent with tweezers at 298 K, as a supplement to Figure 4c.

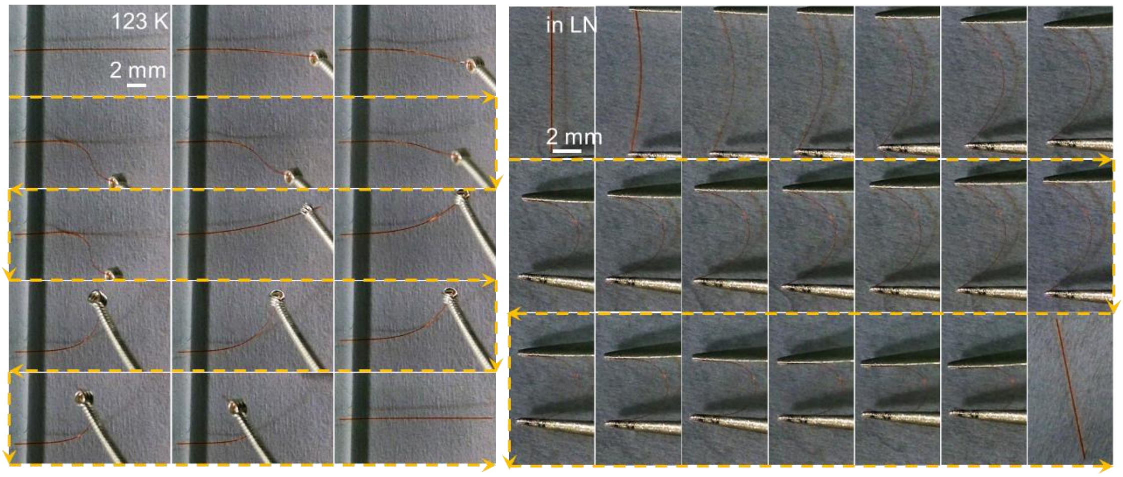

Figure S30. The detailed process of **B10** crystals being repeatedly bent at 123 K and in liquid nitrogen (LN), as a supplement to Figure 4d.

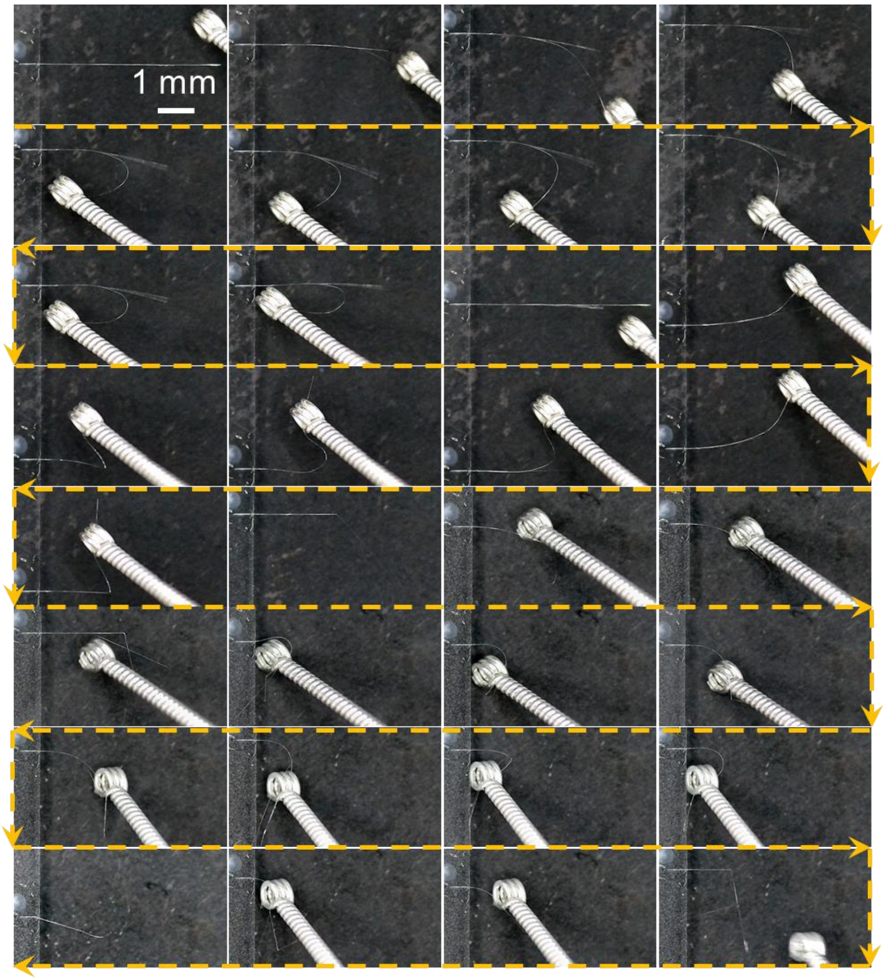

Figure S31. The detailed process of repeated elastic bending and plastic deformation of a **PB10** crystal at 123 K, as a supplement to Figure 4e.

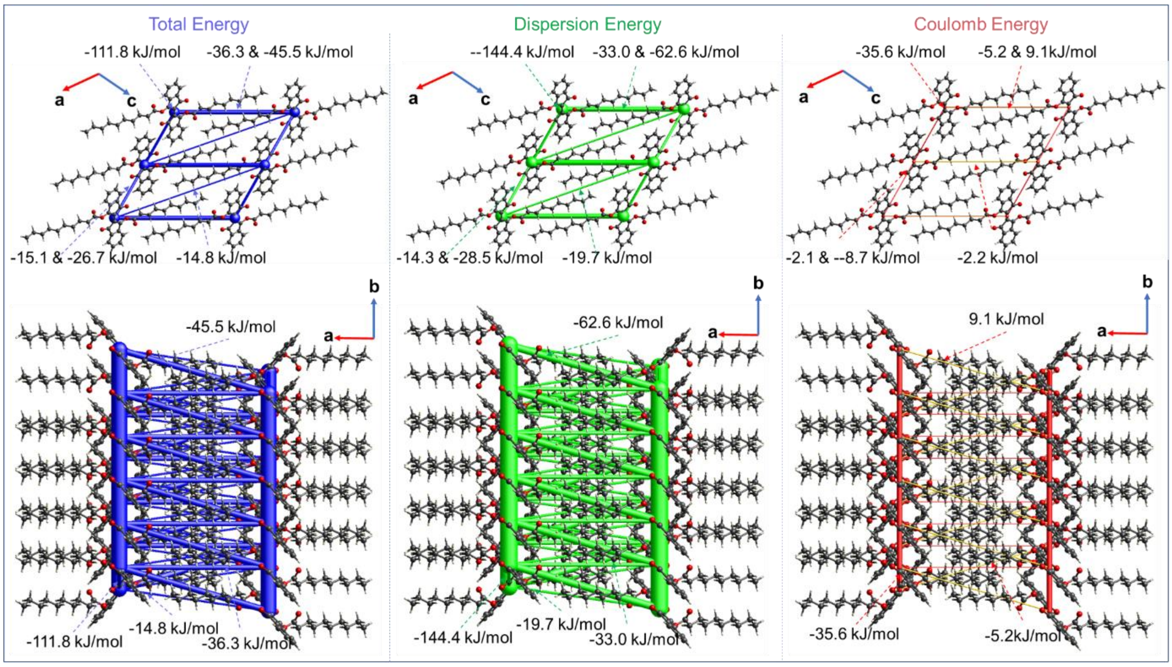

Figure S32. Energy frameworks and magnitudes of different types of interaction energies for the **B10** crystal perpendicular to the *b* and *c* axes, with a 100 energy scale factor and a zero energy threshold.

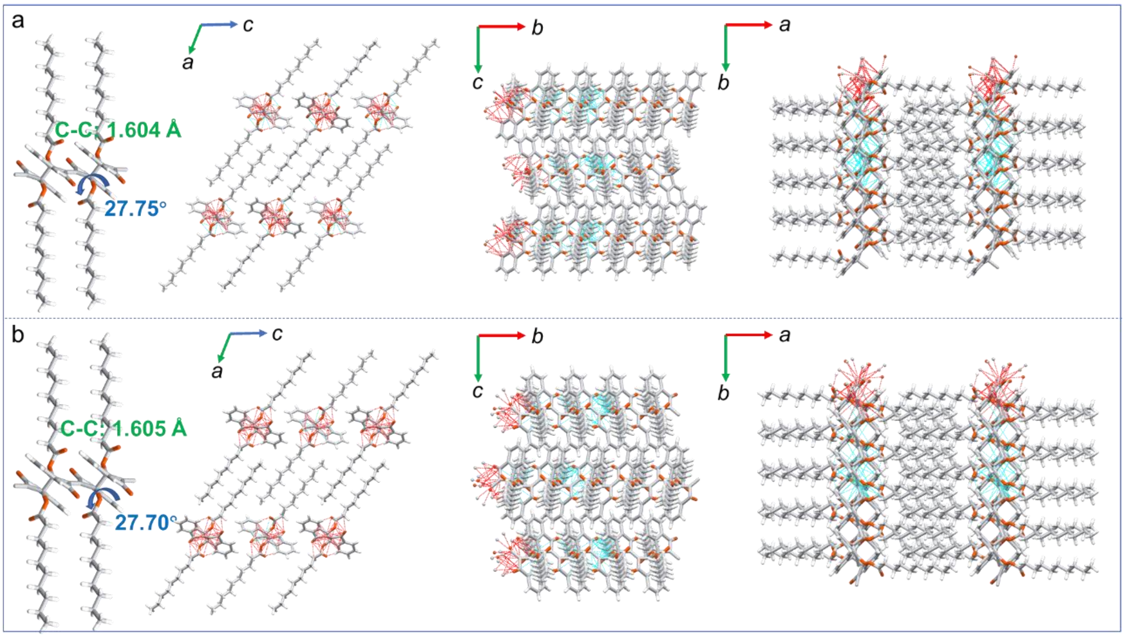

c

PB10	Torsion angle	C-H···O	C-O···C	C-O···O	Other weak interaction
298 K	27.75	2.325 Å	2.685 Å	2.649 Å	2.391~3.363 Å
100 K	27.70	2.306 Å	2.694 Å	2.652 Å	2.390~3.363 Å

Figure S33. (a,b) Comparison of single-crystal structures and stacking modes of **PB10** at 100 K (a) and 298 K (b). (c) Comparison of the torsion angle and weak interactions of **PB10** at different temperatures.

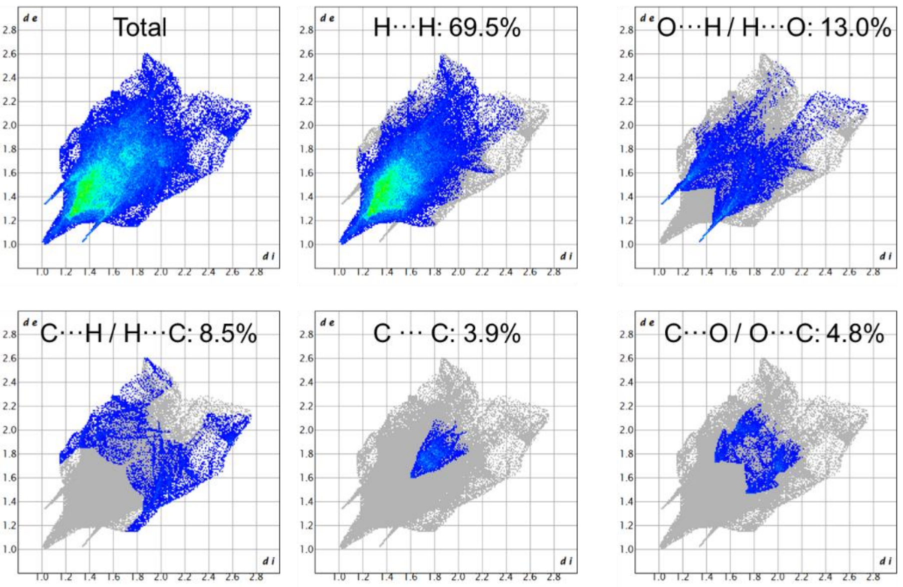

Figure S34. Fingerprint plot for the single molecule of **B10**, indicating features characteristic of the key intermolecular contacts.

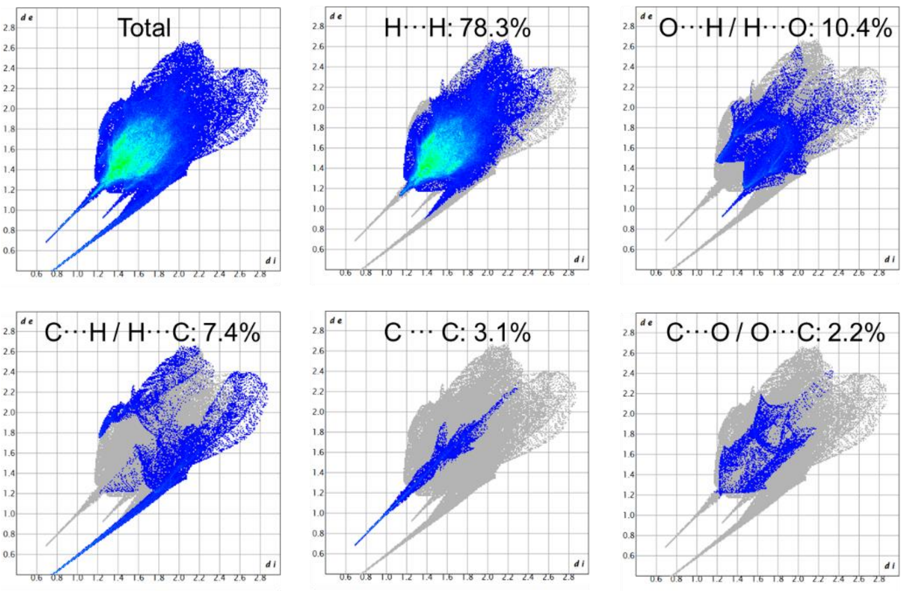

Figure S35. Fingerprint plot for the trimer of **PB10**, indicating features characteristic of the key intermolecular contacts.

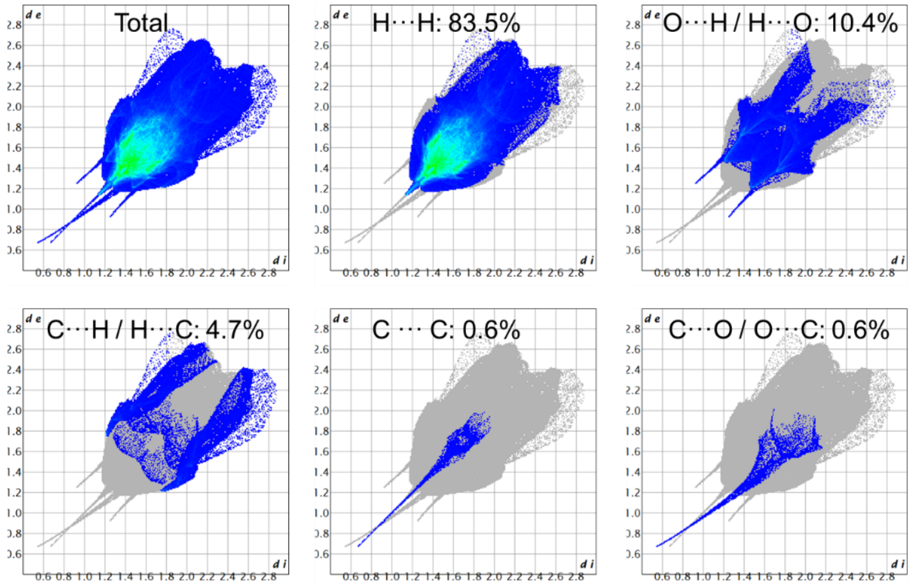

Figure S36. The fingerprint plot for the decamer of **PB10**, indicating features characteristic of the key intermolecular contacts.

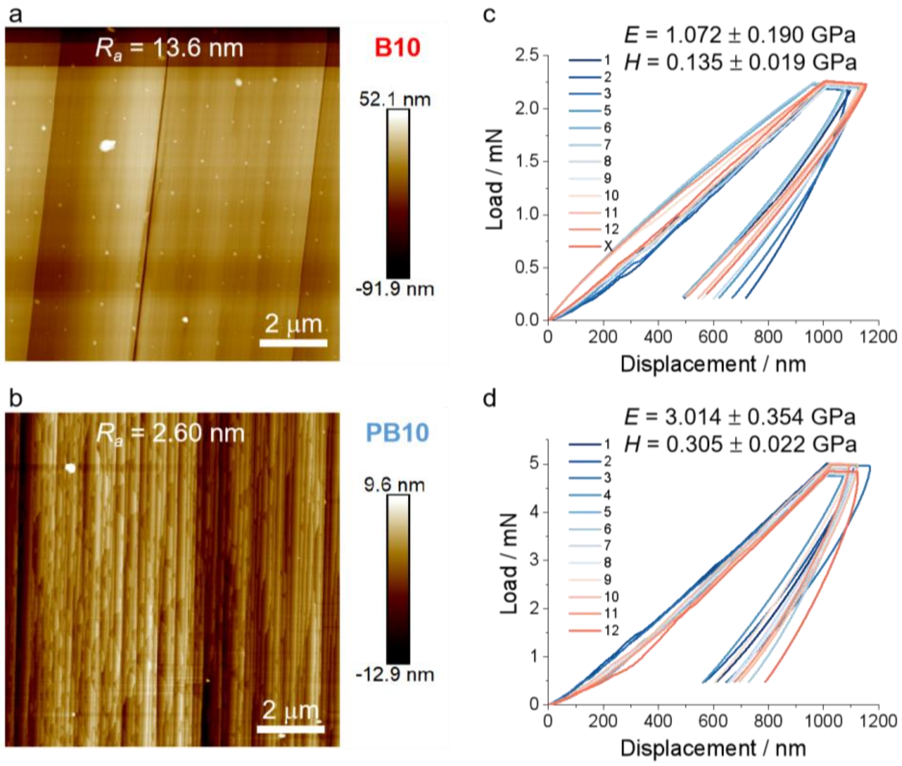

Figure S37. (a,b) AFM images of **B10** (a) and **PB10** (b) crystals showing surface roughness. (c,d) Load-displacement curves of **B10** (c) and **PB10** (d) crystals obtained by nanoindentation.

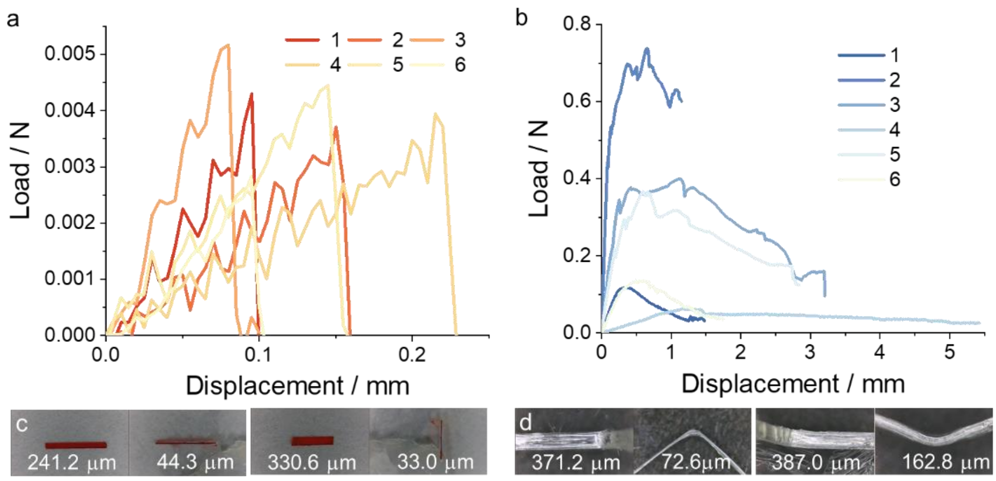

Figure S38. (a,b) Load-displacement curves of **B10** (a) and **PB10** (b) crystals obtained by three-point bending tests. (c,d) Photographs of **B10** (c) and **PB10** (d) crystals after testing.

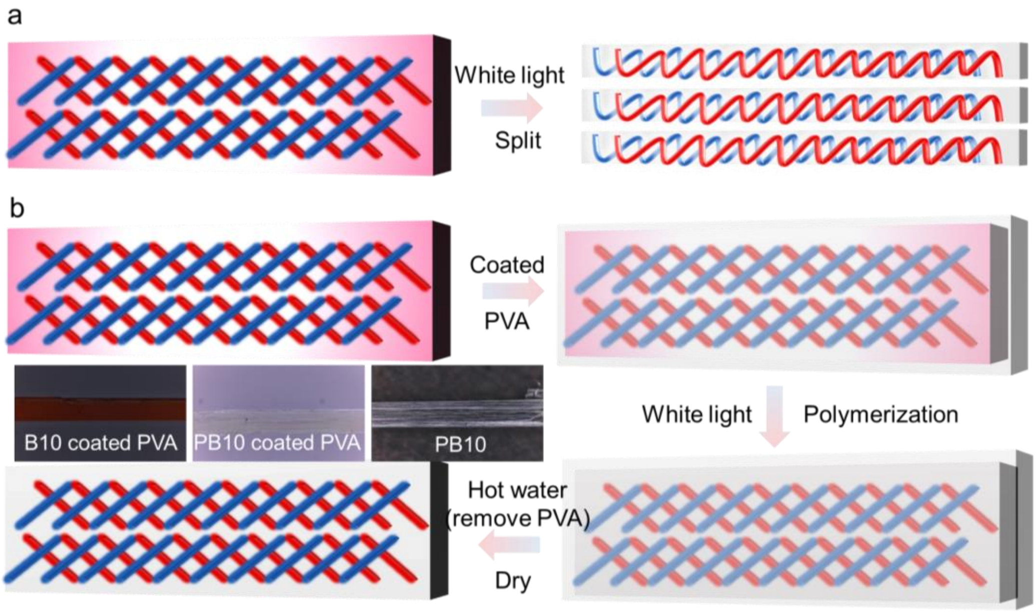

Figure S39. (a) Schematic diagram illustrating the splitting of the original **B10** crystal into multiple **PB10** crystal fragments by irradiation with light. (b) Schematic diagram showing the process for preparation of **PB10** crystals without splitting. The inset images depict the **B10** crystal encapsulated in PVA, which undergoes photopolymerization to form **PB10** crystals still encapsulated in PVA, followed by the removal of PVA to obtain the intact **PB10** crystal.

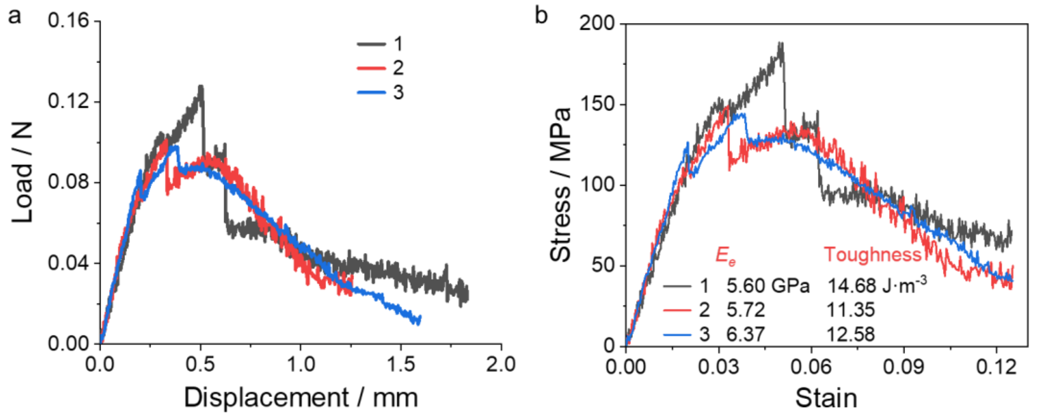

Figure S40. (a,b) Load-displacement (a) and stress-strain (b) curves of different regions (labeled 1–3) of a **PB10** crystal.

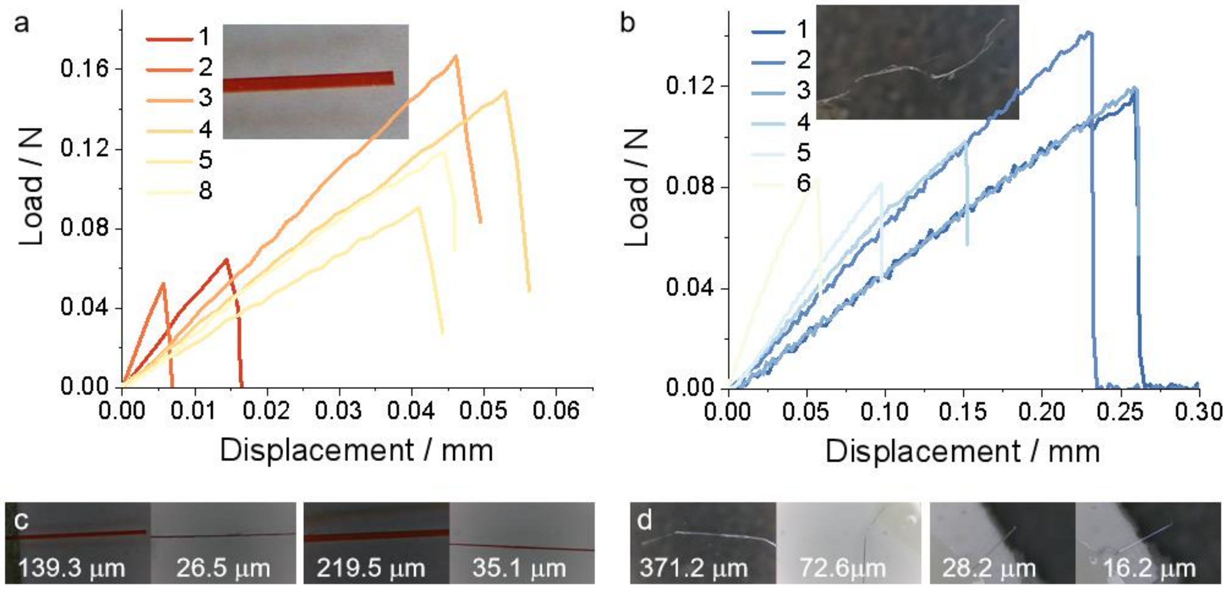

Figure S41. (a,b) Load-displacement curves of **B10** (a) and **PB10** (b) crystals obtained from the tensile tests. The inset images show the fracture morphologies of **B10** and **PB10** crystals. (c,d) Photographs of **B10** (c) and **PB10** (d) crystals after testing.

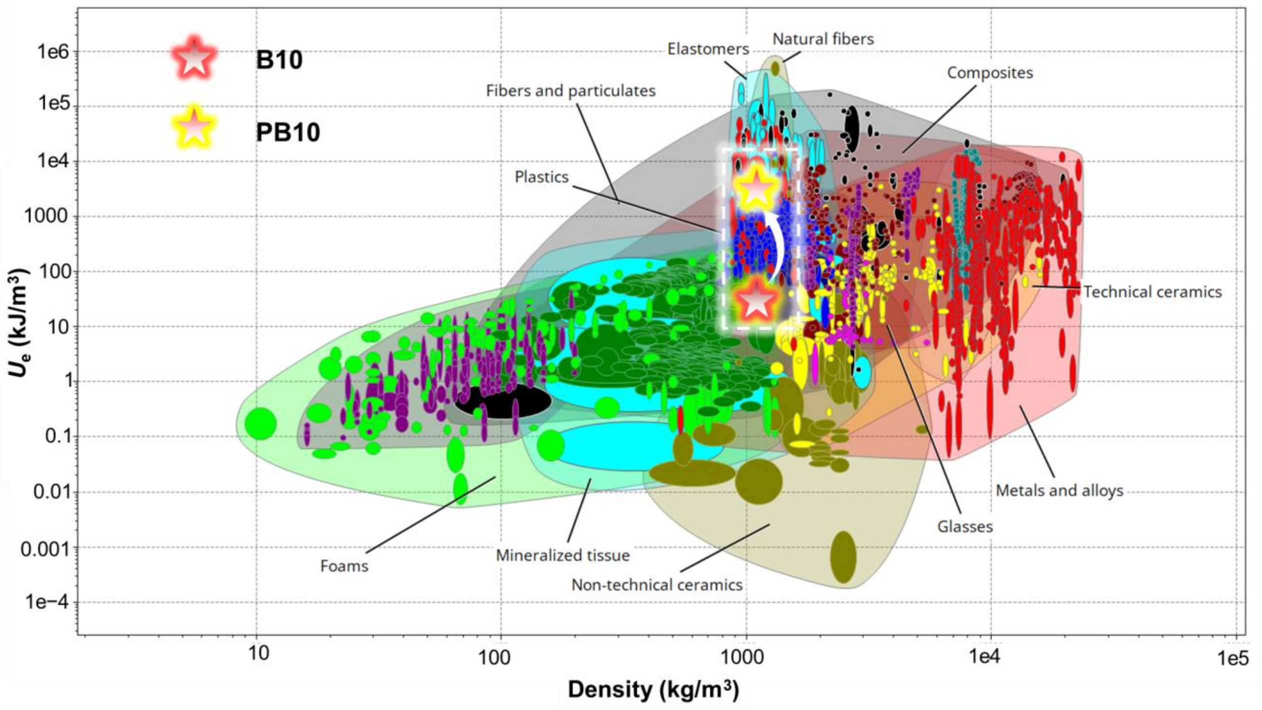

Figure S42. Global materials property plot of toughness (U_t, kJ/m³) against density (kg/m³) for **B10** and **PB10** crystals, positioned relative to common materials such as foams, plastics, composites, technical ceramics, and metals. The polymerized **PB10** exhibits significantly higher elastic stored energy compared to **B10**, demonstrating its enhanced mechanical toughness and energy absorption capabilities. These plots were generated using our own data for the target compounds and comparative datasets obtained via the Granta Selector 2024 software (ANSYS).

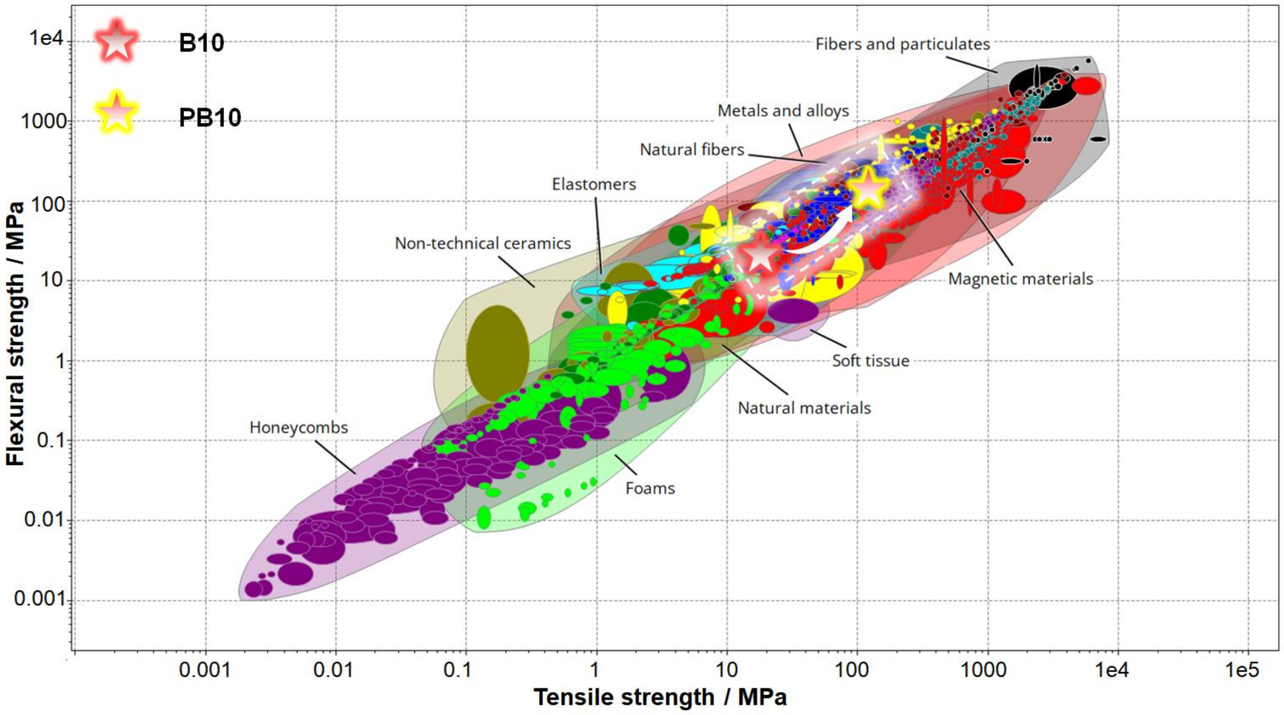

Figure S43. Global materials property plot of flexural strength (modulus of rupture, MPa) against tensile strength (MPa) for **B10** and **PB10** crystals, benchmarked against various material classes, including elastomers, composites, natural fibers, and technical ceramics. **PB10** demonstrates enhanced strength and ductility compared to **B10**, aligning closer to high-performance composites and metals, showcasing the significant gain in mechanical robustness by photopolymerization. These plots were generated using our own data for the target compounds and comparative datasets obtained via the Granta Selector 2024 software (ANSYS).

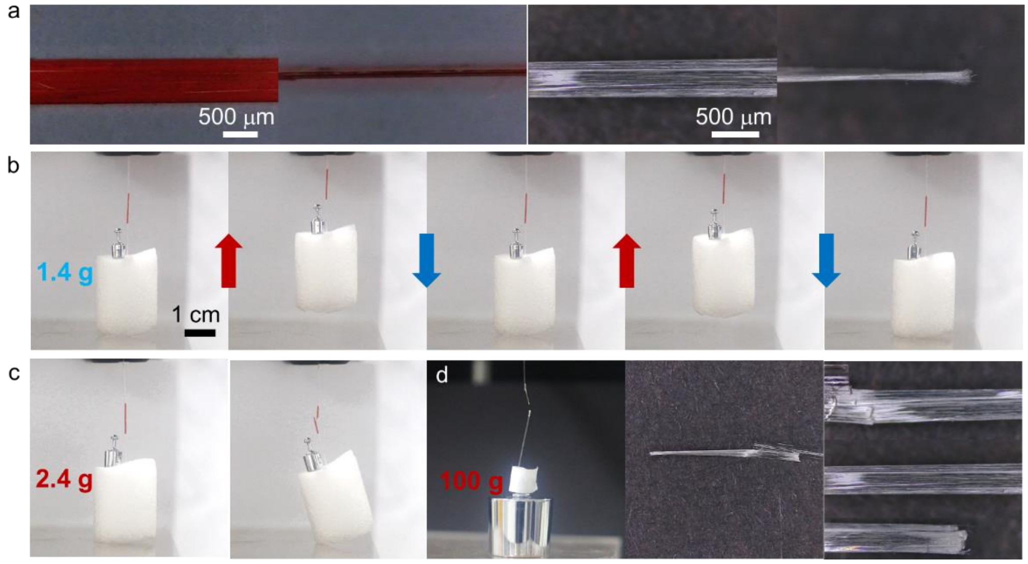

Figure S44. (a) Photographs of **B10** and **PB10** crystals used for measuring load-bearing performance. (b,c) The process of the **B10** crystal lifting of a 1.4 g object (400 mg foam + 1 g weight) and fracturing during repeated lifting of a 2.4 g object (400 mg foam + 2 g weight). (d) Photograph showing the fracture of **PB10** crystals while lifting a 100 g weight, along with a magnified view of the crystal.

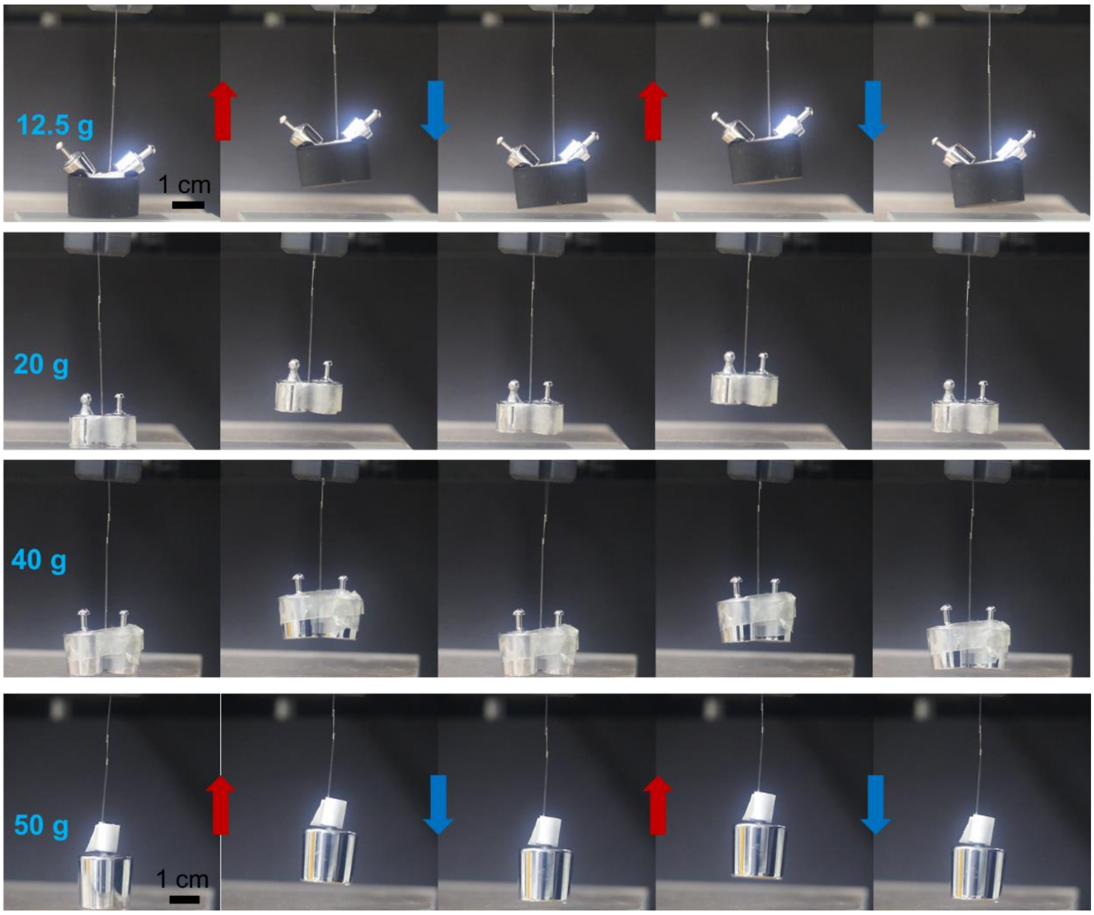

Figure S45. Repeated lifting of weights of varying masses by using the polymer crystal.

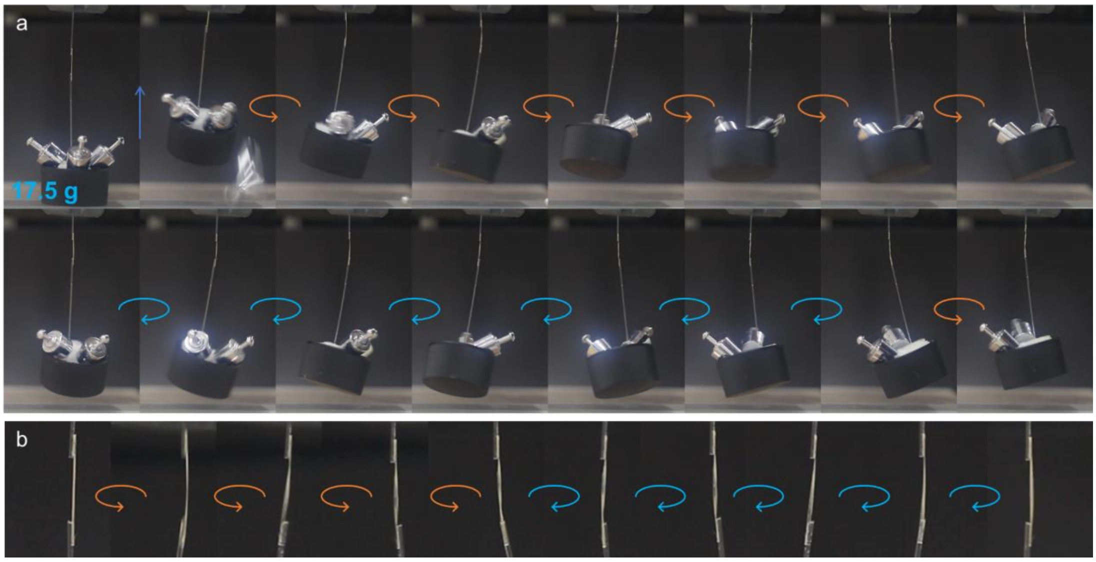

Figure S46. (a,b) Crystal twisting that occurs while lifting heavy weights (a), along with a magnified view (b) showing that the crystal remains intact and does not fracture.

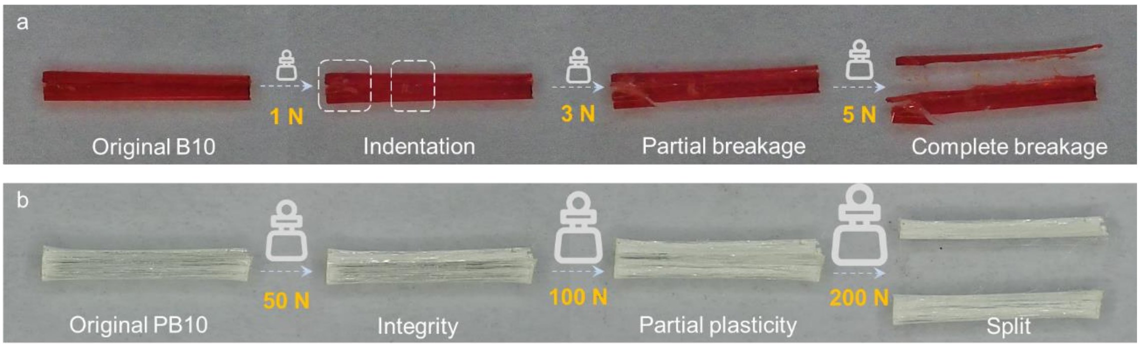

Figure S47. Comparative pressure-bearing performance of **B10** and **PB10** crystals under increasing applied loads. (a) The pristine **B10** crystal fractures under lower forces (1 N, 3 N, and 5 N). (b) The polymerized **PB10** crystal exhibits significantly enhanced mechanical toughness, withstanding much higher forces (50 N, 100 N, and 200 N) before breaking.

3. Supplementary tables

Table S1. Comparison of different-sized samples under white light irradiation: number of fractures, time required to reach maximum deformation (T_d), time to straighten (T_s), and time for complete polymerization (T_p)

Sample	Length / cm	Width / μm	Thickness / μm	Number of fractures	T_d / s	T_s / s	T_p / s
1	1	395	57.85	61	144	174	20
2	1	342.45	40.66	50	112	147	15
3	1	233.29	31.24	29	86	123	11
4	1	135.29	26.59	18	49	89	7
5	1	60.93	16.22	6	32	66	5
6	1	34.99	13.84	4	18	45	4

Table S2. Crystal data and structure refinement for **B10** and **PB10** at 298 K (note that due to the sensitivity of the of crystal **B10** to X-rays at room temperature, only the unit cell parameters could be obtained)

Identification code	**B10**	**PB10**
Empirical formula	$C_{38}H_{46}O_6$	$C_{38}H_{46}O_6$
Formula weight	598.75	598.75
Temperature / K	298	298
Crystal system	monoclinic	monoclinic
Space group	$P2_1/c$	$P2_1/c$
a / Å	19.6229(4)	18.9530(7)
b / Å	4.9393(3)	4.86390(10)
c / Å	19.7131(7)	19.3424(6)
α / °	90	90
β / °	117.461(6)	114.088(2)
γ / °	90	90
Volume / Å^3	1695.32(9)	1627.82(9)
Z	—	2
ρ_{calc} g/cm^3	—	1.222
μ / mm^{-1}	—	0.648

$F(000)$	—	644.0
Crystal size / mm^3	0.2 × 0.1 × 0.2	0.12 × 0.1 × 0.08
Radiation	—	CuK_α (λ = 1.54178)
2Θ range for data collection / °	—	5.108 to 133.074
Index ranges	—	-22 ≤ h ≤ 22, -4 ≤ k ≤ 5, -20 ≤ l ≤ 23
Reflections collected	—	12282
Independent reflections	—	2860 [R_{int} = 0.0659, R_{sigma} = 0.0450]
Data/restraints/parameters	—	2860/0/201
Goodness-of-fit on F^2	—	1.024
Final R indices [I >= 2σ(I)]	—	R_1 = 0.0438, wR_2 = 0.1348
Final R indices [all data]	—	R_1 = 0.0595, wR_2 = 0.1436
Largest diff. peak/hole / e Å^{-3}	—	0.25/-0.22

Table S3. Crystal data and structure refinement for **B10** and **PB10** at 100 K

Identification code	**B10**	**PB10**
Empirical formula	$C_{38}H_{46}O_6$	$C_{38}H_{46}O_6$
Formula weight	598.75	598.75
Temperature / K	100.15	100.15
Crystal system	monoclinic	monoclinic
Space group	$P2_1/c$	$P2_1$/c
a / Å	19.3861(15)	18.972(2)
b / Å	4.9188(3)	4.8631(4)
c / Å	19.5787(15)	19.344(3)
α / °	90	90
β / °	119.508(3)	114.093(7)
γ / °	90	90
Volume / Å^3	1624.8(2)	1629.2(3)
Z	2	2
ρ_{calc} g/cm^3	1.224	1.221
μ / mm^{-1}	0.081	0.647
$F(000)$	644.0	644.0

Crystal size / mm^3	0.1 × 0.1 × 0.1	0.3 × 0.1 × 0.1
Radiation	MoK_α (λ = 0.71073)	CuK_α (λ = 1.54178)
2Θ range for data collection / °	6.33 to 59.314	5.102 to 133.086
Index ranges	$-26 \le h \le 26$, $-6 \le k \le 6$, $-27 \le l \le 27$	$-22 \le h \le 22$, $-5 \le k \le 5$, $-22 \le l \le 22$
Reflections collected	34944	13143
Independent reflections	4571 [R_{int} = 0.0517, R_{sigma} = 0.0300]	2805 [R_{int} = 0.0830, R_{sigma} = 0.0548]
Data/restraints/parameters	4571/0/200	2805/0/200
Goodness-of-fit on F^2	1.017	1.103
Final R indices [$I >= 2\sigma(I)$]	R_1 = 0.0411, wR_2 = 0.1002	R_1 = 0.0496, wR_2 = 0.1355
Final R indexes [all data]	R_1 = 0.0611, wR_2 = 0.1119	R_1 = 0.0695, wR_2 = 0.1515
Largest diff. peak/hole / e $Å^{-3}$	0.39/-0.26	0.30/-0.25

Table S4. Comparison of dimensions and performance metrics of **B10** and **PB10** crystals obtained by three-point bending tests, including maximum strain, maximum stress, elastic modulus (E_e), and toughness

Crystal	Sample	Width / μm	Thickness / μm	Strain / %	Stress / MPa	E_e / GPa	Toughness / MJ / m^3
**B10**	1	241.20	44.31	0.633	27.28	4.16	0.073
	2	330.60	32.98	0.739	31.35	4.15	0.10
	3	276.72	50.61	0.607	21.87	3.87	0.068
	4	293.08	28.92	0.967	45.43	4.39	0.20
	5	213.34	39.47	0.859	40.17	4.5	0.18
	6	260.24	36.93	0.525	23.98	4.84	0.052
Average values				0.72 ± 0.17	31.68 ± 9.34	4.32 ± 0.33	0.11 ± 0.06
**PB10**	1	371.23	72.63	16.06	181.00	7.23	16.65
	2	387.26	162.30	28.14	215.32	6.16	50.17
	3	421.97	193.27	23.19	152.62	7.32	25.23
	4	345.24	74.81	15.22	194.95	6.44	18.76
	5	418.94	195.25	20.56	137.88	5.78	18.88
	6	461.07	66.48	17.46	199.88	5.75	21.14
Average values				20.10 ± 4.93	180.28 ± 29.63	6.45 ± 0.69	25.14 ± 12.60

Table S5. Comparison of dimensions and performance metrics of **B10** and **PB10** crystals under tensile tests, including maximum strain, maximum stress, tensile modulus (E_t), and toughness

Crystal	Sample	Length / cm	Width / μm	Thickness / μm	Strain / %	Stress / %	E_t / GPa	Toughness / MJ / m^3
	1	2.81	139.28	26.55	0.51	17.42	3.4	0.054
	2	1.35	219.55	35.15	0.42	13.59	3.21	0.036
	3	7.42	332.06	27.50	0.62	18.26	2.96	0.063
	4	8.94	327.01	23.41	0.59	19.46	3.26	0.062
	5	9.37	229.52	29.36	0.44	13.45	3.04	0.033
	6	8.09	296.38	25.10	0.55	15.93	2.87	0.046
Average values					0.52 ± 0.08	16.35 ± 2.48	3.12 ± 0.20	0.049 ± 0.013
	1	6.28	28.95	19.12	4.12	214.64	5.33	4.50
	2	6.59	43.04	15.09	3.51	216.82	6.40	3.99
	3	8.47	31.56	19.24	3.08	195.43	6.61	3.05
	4	4.08	25.29	17.71	3.70	219.25	6.10	4.35
	5	3.11	28.17	16.25	3.13	178.12	5.99	2.75
	6	1.42	29.76	12.46	4.02	225.17	5.62	5.31
Average values					3.59±0.44	208.24±17.85	5.84±0.74	3.99±0.95

Table S6. Comparison of load-bearing capacities of **B10** and **PB10** crystals

Crystal	Length / cm	Width / μm	Thickness / μm	Density / g cm^{-3}	Weight of crystal / mg	Weight of the lifting / mg	Weight ratio
**B10**	1.0	469.23	74.31	1.224	0.4268	1400	3280
**PB10**	1.0	384.24	73.15	1.222	0.3434	50000	145603

Table S7. Comparison of load-bearing capacities of **B10** and **PB10** crystals

Crystal	Length / cm	Width / μm	Thickness / μm	Density / g cm^{-3}	Weight of crystal / mg	Weight of the lifting / mg	Weight ratio
**B10**	1.0	469.23	74.31	1.224	0.4268	1400	3280
**PB10**	1.0	384.24	73.15	1.222	0.3434	50000	145603

Table S8. Load-lifting performance of three additional sets of **B10** monomer and **PB10** polymer single crystals.

Crystal	Samples	Length / cm	Weight of crystal / mg	Weight of the lifting / g	Weight ratio
**B10**	1	1.0	0.1635	0.5	3058
	2	1.0	0.139	0.4	2878
	3	1.0	0.2925	1	3419
**PB10**	1	1.0	0.0466	8	171674
	2	1.0	0.0864	12	138889
	3	1.0	0.1348	25	185460

4. Legends for the supplementary movies

Movie S1. Mechanical effects of crystals of **B10** (samples labeled 1–4) of different sizes, and bending and splitting of crystal of **B10** (sample labeled 5) upon exposure to light.

Movie S2. Phase transition and melting of different samples of **PB10** at 483 K.

Movie S3. Bending of **B10** and **PB10** crystals at 298 K by applying external force.

Movie S4. Bending of **B10** and **PB10** crystals at 123 K or 77 K (submerged in liquid nitrogen) by applying external force.

Movie S5. Comparison of the response of **B10** and **PB10** single crystals to pinching with a needle tip. While the crystal of **B10** fractures readily, the one of **PB10** splinters along its longest axis.

Movie S6. Three-point bending of **B10** and **PB10** crystals using a universal mechanical property testing machine. The crystal of **B10** exhibits elastic bending followed by immediate fracture and splitting into two pieces, while the crystal of **PB10** undergoes both elastic and plastic bending.

Movie S7. Tensile tests of **B10** and **PB10** crystals using a universal mechanical property testing machine. Under tension, **B10** breaks into two pieces, while **PB10** curls.

Movie S8. Comparison of the load-bearing capacity of **B10** and **PB10** crystals. The ends of the crystals are fixed to capillary tubes, with one end of the tube attached to weights. The other end of the crystal is repeatedly lifted to test its load-bearing capacity.

5. Supplementary references

1. Dou, L.; Zheng, Y.; Shen, X.; Wu, G.; Fields, K.; Hsu, W.; Zhou, H.; Yang, Y.; Wudl, F. Single-Crystal Linear Polymers through Visible Light–Triggered Topochemical Quantitative Polymerization. *Science* **2014**, *343* (6168), 272–277. DOI: 10.1126/science.1245875.
2. Wei, Z.; Wang, X.; Seo, B.; Luo, X.; Hu, Q.; Jones, J.; Zeller, M.; Wang, K.; Savoie, B. M.; Zhao, K.; Dou, L. Side-Chain Control of Topochemical Polymer Single Crystals with Tunable Elastic Modulus. *Angew. Chem. Int. Ed.* **2022**, *61* (49), e202213840. DOI: 10.1002/anie.202213840.
3. APEX3, Version 2015.52; Bruker AXS Inc.: Madison, WI, **2015**.
4. Dolomanov, O. V.; Bourhis, L. J.; Gildea, R. J.; Howard, J. A. K.; Puschmann, H. OLEX2: A Complete Structure Solution, Refinement, and Analysis Program. *J. Appl. Crystallogr.* **2009**, *42*, 339–341. DOI: 10.1107/S0021889808042726.
5. Macrae, C. F.; Bruno, I. J.; Chisholm, J. A.; Edgington, P. R.; McCabe, P.; Pidcock, E.; Rodriguez-Monge, L.; Taylor, R.; van de Streek, J.; Wood, P. A. Mercury CSD 2.0 – New Features for the Visualization and Investigation of Crystal Structures. *J. Appl. Crystallogr.* **2008**, *41*, 466–470. DOI: 10.1107/S0021889807067908.
6. Ghosh, M. K.; Mishra, S. B.; Kadambi, U.; Ramamurty, R.; Desiraju, G. R. Designing Elastic Organic Crystals: Highly Flexible Polyhalogenated N-Benzylideneanilines. *Angew. Chem. Int. Ed.* **2015**, *54* (9), 2674–2678. DOI: 10.1002/anie.201410730.
7. Liu, H.; Lu, Z.; Zhang, Z.; Wang, Y.; Zhang, H. Highly Elastic Organic Crystals for Flexible Optical Waveguides. *Angew. Chem. Int. Ed.* **2018**, *57* (28), 8448–8452. DOI: 10.1002/anie.201802020.
8. Dassault Systèmes BIOVIA. *Materials Studio User Guide*; Dassault Systèmes: San Diego, CA, **2019.**
9. Frisch, M. J.; Trucks, G. W.; Schlegel, H. B.; Scuseria, G. E.; Robb, M. A.; Cheeseman, J. R.; Scalmani, G.; et al. *Gaussian 09, Revision D.01*; Gaussian, Inc.: Wallingford, CT, **2009**.
10. Turner, M. J.; McKinnon, J. J.; Wolff, S. K.; Grimwood, D. J.; Spackman, P. R.; Jayatilaka, D.; Spackman, M. A. *CrystalExplorer17*; University of Western Australia: Perth, **2017**.
11. Spackman, M. A.; Jayatilaka, D. Hirshfeld Surface Analysis. *CrystEngComm* **2009**, *11* (1), 19–32. DOI: 10.1039/B818330A.

12. Worthy, A.; Grosjean, A.; Pfrunder, M. C.; Xu, Y.; Yan, C.; Edwards, G.; Clegg, J. K.; McMurtrie, J. C. Atomic Resolution of Structural Changes in Elastic Crystals of Copper(II) Acetylacetonate. *Nat. Chem.* **2018**, *10*, 65–69. DOI: doi.org/10.1038/nchem.2848.
